# Intraspecific variation in thermal acclimation of photosynthesis across a range of temperatures in a perennial crop

**DOI:** 10.1093/aobpla/plw035

**Published:** 2016-07-11

**Authors:** Serge Zaka, Ela Frak, Bernadette Julier, François Gastal, Gaëtan Louarn

**Affiliations:** INRA, UR4 URP3F, BP6, F86600 Lusignan, France

**Keywords:** Farquhar model, homeostasis, intra-specific variability, leaf traits, *Medicago sativa*, photosynthesis, temperature acclimation

## Abstract

Interest in the thermal acclimation of photosynthesis has been stimulated by the increasing relevance of climate change. However, little is known about intra-specific variations in thermal acclimation and its potential for breeding. In this article, we examined the difference in thermal acclimation between alfalfa (*Medicago sativa*) cultivars originating from contrasting origins, and sought to analyze the mechanisms in play. A series of experiments was carried out at seven growth temperatures between 5 and 35 °C using four cultivars from temperate and Mediterranean origin. Leaf traits, the photosynthetic rate at 25 °C (*A*_400_^25^), the photosynthetic rate at optimal temperature (*A*_400_^opt^), the thermal optimum of photosynthesis (*T*_opt_), and the photosynthetic parameters from the Farqhuar model were determined. Irrespective of cultivar origin, a clear shift in the temperature responses of photosynthesis was observed as a function of growth temperature, affecting thermal optimum of photosynthesis, photosynthetic rate at optimal temperature and photosynthetic rate at 25 °C. For both cultivars, *T*_opt_ values increased linearly in leaves grown between 5 and 35 °C. Relative homeostasis of *A*_400_^25^ and *A*_400_^opt^ was found between 10 °C and 30 °C growth temperatures, but sharp declines were recorded at 5 and 35 °C. This homeostasis was achieved in part through modifications to leaf nitrogen content, which increased at extreme temperatures. Significant changes were also recorded regarding nitrogen partitioning in the photosynthetic apparatus and in the temperature dependence of photosynthetic parameters. The cultivars differed only in terms of the temperature response of photosynthetic parameters, with Mediterranean genotypes displaying a greater sensitivity of the maximum rate of Rubisco carboxylation to elevated temperatures. It was concluded that intra-specific variations in the temperature acclimation of photosynthesis exist among alfalfa cultivars, but that Mediterranean genotypes presented no evidence of superior performance at high temperatures.

## Abstract (precis)

Plants acclimate to the thermal regime they experience. We analysed intra-specific variations in the thermal acclimation of photosynthesis in a perennial herbaceous crop by comparing cultivars from contrasting origins grown at a range of temperatures. It was concluded that both temperate and Mediterranean cultivars display strong patterns of thermal acclimation in the 5-40°C range. No evidence of superior performance was found for Mediterranean genotypes at high temperatures.

## Introduction

Because plants cannot move, adaptation of their photosynthetic characteristics is essential to maximize performance at their growth temperature (*T*_growth_) (Berry and Bjorkman 1980; Sage and Kubien 2007; Way and Yamori 2014; Yamori *et al.* 2014). In addition to short-term responses of photosynthesis, the thermal acclimation of the photosynthetic system has long been acknowledged as a phenomenon (Berry and Bjorkman 1980; Mooney *et al.* 1978). It is now seen as a critical process to predict impacts of climate change (IPCC 2013 and infer species adaptation (Gunderson *et al.* 2010; Kattge and Knorr 2007).

Berry and Bjorkman (1980) defined photosynthetic acclimation as ‘environmentally induced changes in photosynthetic characteristics that result in an improved performance under the new growth regime’. The thermal acclimation of photosynthesis has been associated with modifications to several photosynthetic variables (Way and Yamori 2014) which include: (i) shifts in the thermal optimum (*T*_opt_) of photosynthesis toward a new *T*_growth_, (ii) a relative homeostasis of the maximum photosynthetic rate between *T*_growth_ (Cowling and Sage 1998; Gunderson *et al.* 2010) and (iii) altered photosynthetic characteristics measured at 25 °C, such as the ratio between the maximum electron transport rate (*J*_max_) and the maximum rate of Rubisco carboxylation (*V*_cmax_) (Berry and Bjorkman 1980; Leuning 1997, 2002; Medlyn *et al.* 2002). Growth temperature is also known to affect leaf expansion (Louarn *et al.* 2010) and the structure of mature leaves (Bula 1972; Hanson *et al.* 1988; Ku and Hunt 1973). Ultimately, growth effects alter leaf traits, such as the final leaf area (*L*_area_), the specific leaf area (SLA) and the amount of nitrogen per unit area (*N*_a_) (Field and Mooney 1986; Garnier *et al.* 1999; Hikosaka 2004; Reich *et al.* 1998), which are tightly related to photosynthetic capacity (Evans 1989; Yamori *et al.* 2005). These growth responses may in part be adaptive, and contribute to the thermal acclimation of photosynthesis (Onoda *et al.* 2004; Sage and Kubien 2007).

Inter-specific differences in the thermal acclimation of photosynthesis have been studied extensively within each of the different photosynthetic pathways (Sage and Kubien 2007; Yamori *et al.* 2014). Among C_3_ species, significant inter-specific differences have been reported relative to the magnitude of responses (Bunce 2000; Hikosaka *et al.* 2006; Mooney 1980; Yamasaki *et al.* 2002; Yamori *et al.* 2010 , 2014). These differences were to some extent linked to the ecological thermal niches of species. For instance, cold-tolerant herbaceous species displayed a more marked degree of maximal temperature homeostasis and a greater ability to shift *T*_opt_ than cold-sensitive ones (Yamori *et al.* 2010). Among trees, species adapted to habitats with broader seasonal or diel temperature variations have also been reported to have greater capacities for acclimation (Cunningham and Read 2002). The mechanisms underpinning these inter-specific differences have not been fully elucidated. However, differences in the capacity to balance nitrogen partitioning in the photosynthetic apparatus with growth temperature were associated with differences in thermal acclimation among herbaceous species (Hikosaka *et al.* 2006; Onoda *et al.* 2005; Yamori *et al.* 2010). Differences in the ability to adjust the temperature dependencies of photosynthetic reactions limiting CO_2_ assimilation, namely the rates of ribulose-1,5-biphospate (*RuBP*) carboxylation and regeneration, were also identified (Bunce 2000). Finally, differences have been reported in the heat stability of Rubisco activase, resulting in the de-activation of Rubisco (ribulose-1,5-bisphosphate carboxylase/oxygenase) at lower temperatures in species adapted to cold environments (Hikosaka *et al.* 2006; Salvucci and Crafts-Brandner 2004; Sage *et al.* 2008).

By comparison, the study of intra-specific variations in thermal acclimation has received far less attention. A couple of studies showed that differences exist among ecotypes (Bjorkman *et al.* 1975; Ishikawa *et al.* 2007; Mooney 1980; Pearcy 1977). However, little is known about the range of plasticity existing within a species compared to the inter-specific range, in part because most previous studies were limited to comparing two *T*_growth_ only. Furthermore, positive relationships were found between the acclimation potential and the altitude or thermal regime of ecotypes in some (Ishikawa *et al.* 2007), but not all species (Gunderson *et al.* 2000; Teskey and Will 1999). Nevertheless, it is still necessary to determine how intra-specific diversity in terms of photosynthesis acclimation might be related to the ability of a species to occupy a broad range of habitats and achieve this acclimation.

The objectives of this study were to characterize the differences in thermal acclimation between alfalfa (*Medicago sativa*) cultivars originating from contrasting temperate and Mediterranean areas, and to analyse the mechanisms in play. Alfalfa is a temperate perennial forage legume with a broad geographic distribution that ranges from Northern Europe and Canada to North Africa and Florida (Michaud *et al.* 1988). It can generally cope with cold winters, as well as hot dry summers, and could potentially express a broad thermal acclimation of photosynthesis (Yamori *et al.* 2014). The species has been shown to present significant genetic diversity for heat and cold tolerance (McKenzie *et al.* 1988) as well as maximal photosynthesis at 25 °C (Delaney and Dobrenz 1974; Heichel *et al.* 1988). We compared the thermal acclimation of two cultivars and two clones propagated from cuttings at seven growth temperatures between 5 and 35 °C. Thermal acclimation was analysed in terms of leaf traits, thermal optimum, maximum photosynthesis and photosynthetic parameters (*V*_cmax_, *J*_max_ and their temperature dependencies) at each *T*_growth_. The Farquhar model (Farquhar *et al.* 1980) was used to infer the relative importance of the different photosynthetic parameters to the thermal acclimation observed.

## Methods

### Plant materials and growing conditions

A series of experiments was performed in a 8.1 m^2^ growth chamber (model 97132/7NU, Froids et Mesures, Beaucouzé, France) at the INRA Lusignan research station, France. Independent experiments were carried out successively at seven growth temperatures (*T*_growth_), ranging from 5 to 35 °C with 5 °C increments. During each experiment, two alfalfa (*M.* *sativa*) commercial cultivars (Harpe and Barmed from temperate and Mediterranean origin, respectively) and two clones propagated from stem cuttings (G3 and 7_7 clones isolated from temperate and Mediterranean cultivars Orca and Demnate, respectively; Maamouri et al. 2015) were used. Because alfalfa cultivars are synthetic varieties (i.e. populations of half-sibs containing significant genetic diversity; Julier *et al.* 2000), clones were selected in order to replicate identical genotypes in the different experiments. One clone and eight seedlings of each cultivar were replicated four times in a random block design.

The cuttings were produced in a greenhouse about 3 months before each experiment. Seeds of the synthetic varieties were pre-germinated in the dark at 25 °C for 96 h before each experiment. Seedlings and clone cuttings were transplanted individually into 1.5 L pots (10 × 20 cm cylindrical pots) filled with fine quartz (0.8–1.4 mm mesh). The pots were ferti-irrigated with a complete nutrient solution at intervals ranging from three times (5 °C) to eight times a day (35 °C). At the 15 °C *T*_growth_, a problem encountered during the propagation of the cuttings prevented their study.

Two phases were distinguished during each experiment. First, a conditioning period at 25 °C and 70 % relative humidity was applied for 3 weeks. At the end of this period, plant development had achieved five leaves on the main stems of seedlings, and 8–11 leaves on cuttings. The plants were then placed at the studied *T*_growth_ for a period corresponding to five phyllochrones. The air temperature was adjusted to ensure a daily average leaf temperature of *T*_growth_. Day/night temperatures were thus 5/3, 10/8, 15/13, 20/18, 25/23, 30/28 and 35/33 °C at *T*_growth_ of 5, 10, 15, 20, 25, 30 and 35 °C, respectively. The vapour pressure deficit (VPD) was maintained below 1.5 kPa at all *T*_growth_ by adjusting the relative humidity. Lights were set on a 14-/10-h (light/dark) photoperiod (POWERSTAR, HQI-BT 400WD lamps, OSRAM, Munich, Germany). The photosynthetic photon flux density (PPFD) measured at the pot level ranged from 400 to 450 µmol photon m^−2^ s^−1^.

### Gas exchange measurements and determination of photosynthetic parameters

At the end of each experiment, gas exchange measurements were performed using a portable photosynthesis system (LI-6400, Li-Cor, Lincoln, NE, USA) with a 6 cm^2^ (2*3 cm) cuvette. The youngest mature leaf on the main stem (i.e. node ranks 8–9 for seedlings and 12–16 for cuttings) were used for each plant **[Supporting Information Table 1]**.

Light saturated net photosynthesis at ambient CO_2_ (400 ppm, *PPFD* of 1500 µmol photon m^−2^ s^−1^) and dark respiration were measured at 25 °C (*A*_400_^25^) and *T_growth_* (*A*_400_^growth^) on four (clones) or eight (seedlings) plants per cultivar. In addition, the responses of *A* to internal CO_2_ concentration (*C*_i_) at the substomatal level (*A–C*_i_ curves) were determined on the cuttings leaves. Different levels of *C*_i_ were obtained by modifying the ambient CO_2_ concentration (*C*_a_) in the leaf measurement chamber. The *A-C*_i_ curves were compiled as proposed by Long and Bernacchi (2003). First, the value of *A* at the actual *C*_a_ level was recorded, and then *C*_a_ was gradually reduced to five different levels below the ambient concentration. Thereafter, *C*_a_ was returned to the initial value and increased to seven different levels up to 1500 µmol.^mol^^−1^. Each *C*_a_ step was maintained for 5 minutes in order to record stable values. All curves were compiled at 1500 µmol m^−^^2^ s^−2^ *PPFD*, the leaf temperature was controlled at 25 °C and the VPD between the leaf and the air was kept at 1 ± 0.5 kPa. The photosynthetic parameters (*V*_cmax_ and *J*_max_) were estimated simultaneously by fitting the biochemical model developed by Farquhar *et al.* (1980) to the whole *A–C*_i_ curve, according to the procedure proposed by Sharkey *et al.* (2007). Overall, *A–C*_i_ curves and photosynthetic parameters were determined at five different leaf temperatures (*T*_growth_, 10, 25, 35 and 42 °C) for each clone (G3 and 7_7) and each growth temperature.

### Leaf traits

Immediately after the gas exchange measurements, the leaves were scanned (Konica Minolta C352/C300, Konica Minolta Sensing, Osaka, Japan). The leaf area (*L*_area_) was determined by image analysis (ImageJ software, http://rsbweb.nih.gov/ij/, last accessed 31 May 2016). The leaves were then dried at 60 °C for 2 days, weighed to determine their dry mass and ground in a vibrating ball mill (MM400, Retsch GmbH and Co, Haan, Germany). Leaf samples were analyzed with an elemental analyser (model EA 1108, Carlo Erba Instruments, Milan, Italy) to determine their N concentration. The SLA (m^2^ g^−1^) and leaf nitrogen content per unit of area (*N*_a_, g N m^−2^) were then calculated.

### Thermal optimum and determination of the temperature dependencies of photosynthetic parameters

The response curves were fitted to temperature using the *nls* procedure under R software (R Development Core Team 2005). The thermal optimum and response to temperature of light saturated net photosynthesis at 400 ppm CO_2_ were determined using a beta function (Equationn 1, Yan and Hunt 1999):

(1) $$A_{400}=A_{400}^{\text{opt}}\cdot \left[(\frac{T-T_{\min}}{T_{\text{opt}}-T_{\min}})\cdot {\left(\frac{T_{\max}-T}{T_{\max}-T_{\text{opt}}}\right)}^{\frac{T_{\max}-T_{\text{opt}}}{T_{\text{opt}}-T_{\min}}}\right]$$

The temperature dependencies of *V*_cmax_ and *J*_max_ were fitted using the Arrhenius model if there was an exponential increase with temperature (Equation 2), or using a modified Arrhenius function if a significant decline was measured at high leaf temperatures (Equation 3.):

(2) $$r=\frac{\text{exp}\left[C-(\frac{\Delta H_{\text{a}}}{R.T_{1}})\right]}{P^{25}}$$

(3) $$r=\frac{\frac{\text{exp}\left[C-\left(\frac{\Delta H_{\text{a}}}{R.T_{1}}\right)\right]}{1+\text{exp}\left[\left(\frac{\Delta S}{R}\right)-\left(\frac{\Delta H_{a}}{R.T_{1}}\right)\right]}}{P^{25}}$$

where *r* is the rate normalized by the parameter value at 25 °C, *C* is equal to 1 + exp[(*ΔS.T_0_* *−ΔH*_d_)/(*R.T*_0_)] (no dimension), *T*_1_ is the leaf temperature.

### Determination of the limiting steps of photosynthesis

The model developed by Farquhar *et al.* (1980) was used to infer the limiting steps of photosynthesis in the response of *A* to temperature under the different combinations of genotypes and growth temperatures studied. The version implemented on the OpenAlea Modelling Platform was used (Prieto *et al.* 2012) with the default parameters for alfalfa (Louarn *et al.* 2015). All the variables, parameters and symbols used are detailed in Appendix 1. The Farquhar model assumes that the net photosynthetic rate is limited by either the activation state, quantity and kinetic properties of Rubisco (*A*_c_) or *RuBP* regeneration in the Calvin cycle (*A*_r_):

(4) $$A_{r}=\frac{V_{c\max}\;\cdot C_{i\;}}{C_{i\;}+\;K_{c}\;\cdot \left(1\;+\;\frac{O}{K_{o}}\right)}.\left(1-\frac{\Gamma^{*}}{C_{i}}\right)-R_{\text{d}}$$

(5) $$A_{r}=\frac{J\cdot C_{i}}{4\;\cdot {\text{C}}_{i}\;+\text{ 8 }\cdot \Gamma \text{*}}.\left(1-\frac{\Gamma^{*}}{C_{i}}\right)-R_{\text{d}}$$

It was assumed that the kinetic properties of Rubisco (*K*_c_, *K*_o_, and Γ* which depend on the specificity factor of the Rubisco for CO_2_ and O_2_) were constant between genotypes and growth temperatures (Bernacchi *et al.* 2001; Harley *et al.* 1992; Sharkey *et al.* 2007).

*C*_i_ was calculated from measured *C*_a_ and leaf temperature by coupling the semi-empirical stomatal conductance model proposed by Ball et al. (1987) to the Farquhar model. *R*_d_ was calculated from measured values of dark respiration at 25 °C (*R*_d_^25^) and an Arrhenius function accounting for its temperature dependence. The set of gas exchange parameters estimated by Louarn et al. (2015) was used.

For each genotype and *T*_growth_, the photosynthetic parameters and their temperature dependencies were used to examine the relationship between changes in the *A*_400_ response to temperature and changes in parameter values. First of all, simulations were performed using the parameters actually measured in each situation. Then, three series of simulations were conducted to assess the sensitivity of *T*_opt_ and the overall temperature response shape to photosynthetic parameters: (i) assuming a constant ratio between *J*_max_^25^ and *V*_cmax_^25^, (ii) assuming unchanged temperature dependencies for *V*_cmax_ and *J*_max_ and (iii) assuming both a constant ratio and unchanged temperature dependencies.

### Statistical analyses

Statistical analyses were performed using R software (R Development Core Team 2005). Analyses of variance (ANOVA, *aov* procedure) were used to test for significant differences between means and assess effects of temperature treatments and genotype on the values of leaf traits (*L*_area_, SLA and *N_a_*), assimilation rates (*A*_400_^25^) and photosynthetic parameters (*V*_cmax_ and *J*_max_). Multiple comparison tests were performed using the LSD procedure. Linear regression lines were fitted to the data using the *lm* procedure. Linear relationships were tested between observed and predicted *A*_400_ and *T*_opt_ values and were compared with the theoretical 1:1 line. In addition, the model error (RMSE) and bias (*Bias*) were calculated as follow:

(6) $$\text{RMSE }=\;\sqrt{\frac{\sum_{i=1}^{n}{\left(s_{i}-m_{i}\right)}^{2}}{n}}$$

(7) $$\text{Bias }=\;\frac{\sum_{i=1}^{n}\left(s_{i}-m_{i}\right)}{n}$$

## Results

### Impact of growth temperature on leaf growth and leaf nitrogen content

**Figure 1.**
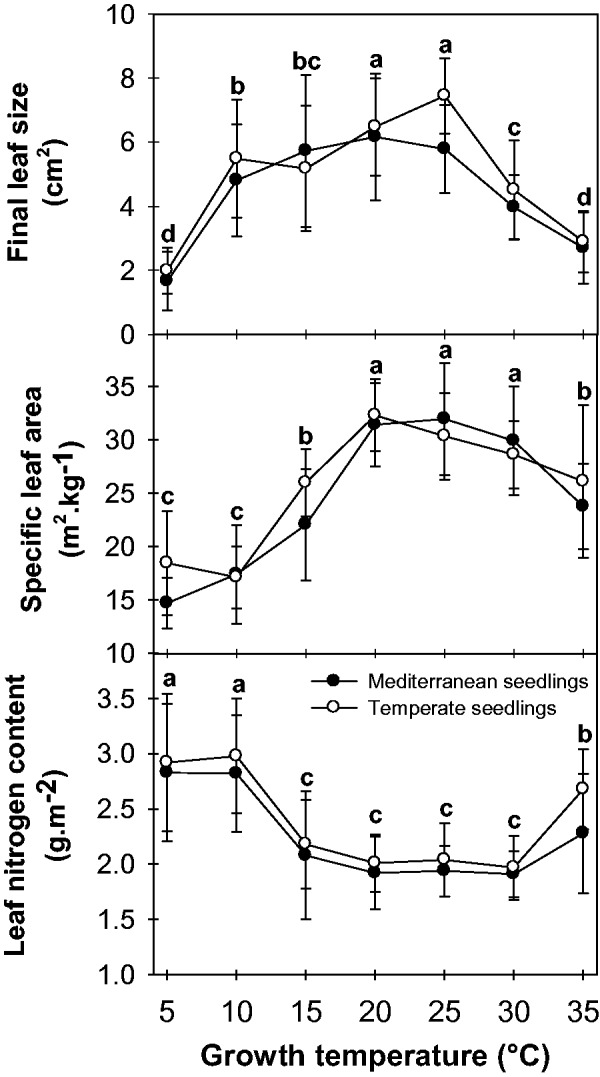
Impact of growth temperature on the final leaf size, SLA and leaf nitrogen concentration of two alfalfa cultivars of temperate (open circles, Harpe cv) and Mediterranean (filled circles, Barmed cv) origins. Leaves were all sampled on the main stem between node ranks 8 and 9. The letters indicate homogeneous groups of means between the different growth temperatures (Fisher’s LSD test). No significant cultivar effect was found.

The impacts of growth temperature (*T*_growth_) on final leaf size (*L*_area_), SLA and leaf nitrogen content (*N*_a_) are presented in Figure 1. Growth temperature significantly affected the three leaf traits studied (ANOVA, *F*_6,42 _>_ _5.9, *P* < 10^−^^3^) in both seedling (Fig. 1) and cutting (not shown) plants. The final leaf size was highest at intermediate *T*_growth_ (20 and 25 °C) and was smaller at both ends of the temperature range tested (5 and 35 °C). SLA was also maximum at a moderate *T*_growth_ (20–30 °C). The reduction in SLA due to low *T*_growth_ (5 and 10 °C) appeared to be greater than that observed at high temperatures (35 °C) within the range tested. Concerning the nitrogen content, *N*_a_ patterns mirrored the pattern observed for SLA. *N*_a_ was lowest at a moderate *T*_growth_ (15–30 °C) and maximum at extreme *T*_growth_ values (5, 10 and 35 °C). ANOVA analyses did not demonstrate any significant differences regarding the origins of the plants in terms of *L*_area_ and SLA (ANOVA, *F*_1,85 _<_ _29.0, *P* > 0.2), but were significant for *N*_a_ (ANOVA, *F*_1,85 _=_ _17.7, *P* < 0.05).

At a *T*_growth_ of 35 °C, symptoms of thermal stress became apparent in the form of heat-bleached leaves on some plants from the Mediterranean cultivars, but never on plants from temperate cultivars (see **[Supporting Information Figure 1]**). These symptoms occurred at a frequency of about 30% in the Barmed cv. and were observed on the 7_7 clone.

### Impact of growth temperature on the net assimilation rate at 25 °C

**Figure 2.**
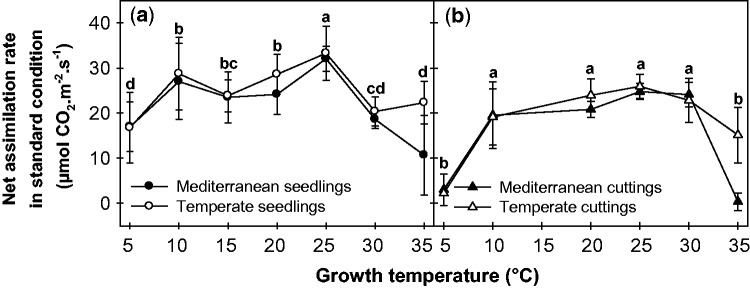
Impact of growth temperature on the net rate of light-saturated photosynthesis measured at 25 °C and 400 ppm CO_2_ (*A*_400_^25^) on seedlings (a) and cuttings (b) of alfalfa of Mediterranean (filled symbols) and temperate (open symbols) origins. Measurements were performed on the youngest mature leaf of the main stem at the end of the experiment (i.e. node ranks 8–9 for seedlings and 12–16 for clones). The letters indicate homogeneous groups of means between the different growth temperatures (Fisher’s LSD test).

The impact of growth temperature on the net rate of light saturated photosynthesis measured under standard conditions (i.e. at a leaf temperature of 25 °C, *A*_400_^25^) is presented in Figure 2. A significant effect of *T*_growth_ on *A*_400_^25^ was observed in both seedlings and cuttings from temperate and Mediterranean origins (ANOVA, *F*_6,42 _>_ _6.1, *P* < 10^−^^3^). *A*_400_^25^ remained relatively constant over a broad range of *T*_growth_ between 10 and 30 °C. However, a drop at extreme *T*_growth_ values (5 and 35 °C) was observed for both alfalfa cultivars and cuttings. The different plant materials displayed similar response patterns, but with slight differences between seedlings and cuttings. In either plant materials, Mediterranean and temperate origins did not affect *A*_400_^25^ between 5 and 30 °C (ANOVA, *F*_1,69 _<_ _1.5, *P* > 0.21). Mediterranean plants displayed a decreased photosynthetic capacity at the 35 °C growth temperature (*P* < 0.02).

### Acclimation of net assimilation to growth temperature

**Figure 3.**
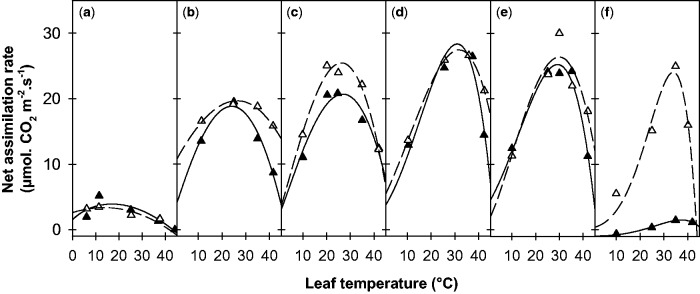
Temperature responses of the net assimilation rate at ambient CO_2_ (*A*_400_) for growth temperatures of 5 °C (a), 10 °C (b), 20 °C (c), 25 °C (d), 30 °C (e) and 35 °C (f). Data are presented for two alfalfa cuttings of Mediterranean (filled triangles, 7_7 cutting) and temperate (open triangles, G3 cutting) origins. Lines represent fits of Equation (1). Each point is the average of measurements on three to four mature leaves (node ranks 12–16).

The responses of *A*_400_ to leaf temperature are presented at the different growth temperatures in Figure 3. A clear shift in the temperature responses of photosynthesis was observed as a function of *T*_growth_, affecting both the optimal temperature (*T*_opt_) and the net assimilation rate at *T_opt_* (*A*_400_^opt^). Over the whole range of growth temperatures, *T*_opt_ (as determined by fitting a beta function, Equation 1) rose regularly from about 18 °C (for leaves grown at 5 °C) to 35 °C (for leaves grown at 35 °C). An acclimation of the response curve thus occurred in the two genotypes studied, which tended to maximize photosynthetic rates within a temperature range close to the growth temperature. However, *A*_400_^opt^ did not remain constant in response to temperature (ANOVA, *F*_5,13_ > 7.3, *P* < 10^−2^). The maximum rate peaked for leaves grown at 25 °C and was significantly reduced with *T*_growth_ below 15 °C and above 30 °C. A marked difference in the maximum rates of the two genotypes was observed at a *T*_growth_ of 35 °C, but not with the other *T*_growth_. Significant differences were also observed between the two genotypes relative to photosynthetic rates at high *T*_leaf_ (42 °C), the Mediterranean genotype displaying an accentuated decrease at 10, 25 and 30 °C *T*_growh_ (ANOVA, *F*_1,10 _>_ _4.2, *P* < 0.05). This resulted in slightly altered shapes of the response curves.

### Impact of growth temperature on photosynthetic parameters and their responses to leaf temperature

**Figure 4.**
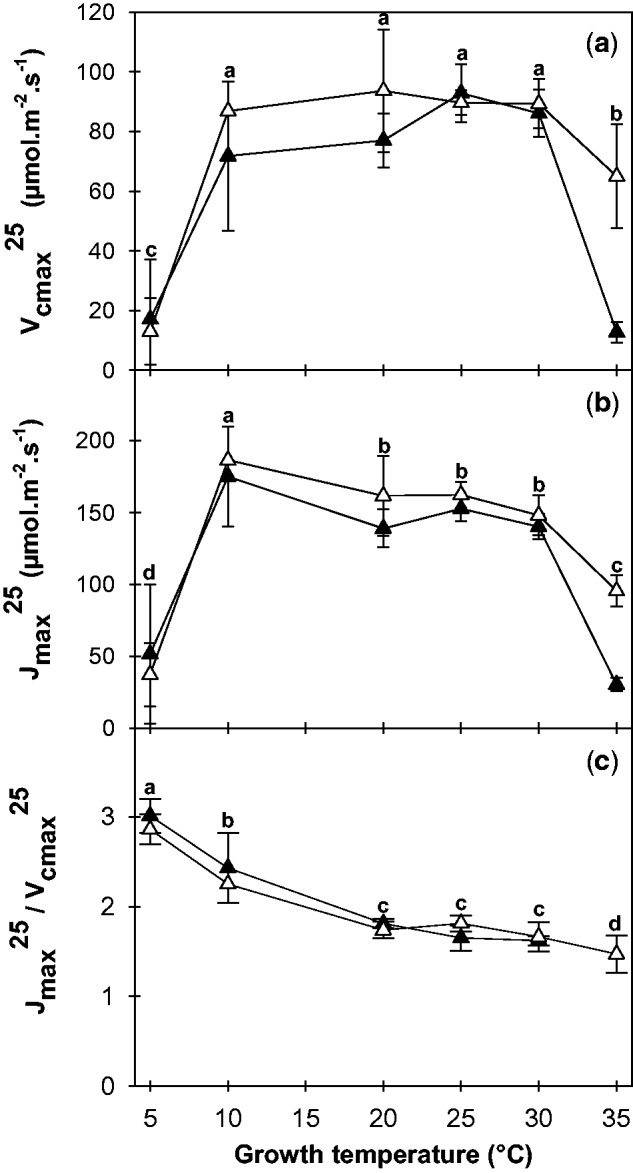
Impact of growth temperature on the maximum catalytic rate of Rubisco (*V*_cmax_^25^, a), the maximum electron transport rate (*J*_max_^25^, b) and their ratio (c, *J*_max_^25^/*V*_cmax_^25^) for two alfalfa genotypes of Mediterranean (filled triangles, 7_7) and temperate (open triangles, G3) origins. The letters indicate homogeneous groups of means between the different growth temperatures (Fisher’s LSD test).

The photosynthetic parameters determined under standard conditions are presented in Figure 4 at the different growth temperatures. The *V*_cmax_^25^ and *J*_max_^25^ parameters were significantly affected by *T*_growth_ (ANOVA, *F*_5,12 _>_ _15.9, *P* < 10^−^^3^). *V*_cmax_^25^ values remained constant between 10 and 30 °C, but were lower at 5 and 35 °C. The values of *J*_max_^25^, on the other hand, decreased between 10 and 30 °C and presented a relatively more limited drop at 5 °C. Overall, the *J*_max_^25^*/V*_cmax_^25^ ratio fell regularly for leaves grown at between 5 and 35 °C (Fig. 4c). No significant differences between genotypes from temperate and Mediterranean origins were observed (ANOVA, *F* =1.4, *P* > 0.29), except at 35 °C where the declines in both *V*_cmax_^25^ and *J*_max_^25^ were more pronounced in the Mediterranean genotype (heat-bleached leaves).

**Figure 5.**
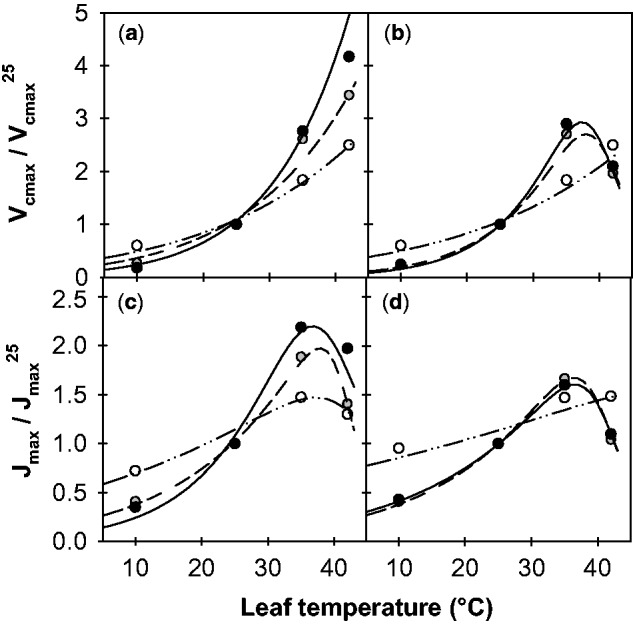
Temperature dependencies of the maximum rate of Rubisco carboxylation (*V*_cmax_, a and b) and maximum electron transport rate (*J*_max_, c and d) of alfalfa leaves grown at different temperatures. Data are given for two genotypes of Mediterranean (b–d, 7_7) and temperate (a–c, G3) origins at growth temperatures of 5 °C (open circles), 25 °C (grey circles) and 30 °C (filled circles). Normalizations by the rates at 25 °C were performed. The lines represent fits of either Equations (2) or (3), the parameters of which are given in **[Supporting Information Table 1].**

At each *T*_growth_, the dependencies of photosynthetic parameters on leaf temperature were also determined. The parameters of these response curves are summarized in **[Supporting Information Table 1].** Figure 5 presents three examples of these curves at contrasting growth temperatures for the two cuttings studied. The responses of *V*_cmax_ and *J*_max_ to leaf temperature were modified by growth temperature in both genotypes. The magnitude of the normalized responses increased with the rise in growth temperature for both *V*_cmax_ and *J*_max_. In addition, changes to the response curves differed between the temperate and Mediterranean genotypes. *V*_cmax_ displayed a typical increasing Arrhenius response curve (Equation 2) irrespective of *T*_growth_ in the temperate genotype, whereas a shift toward an optimum curve (best fitted by a Johnson function, Equation 3) was observed in the Mediterranean genotype grown at 25 and 30 °C. Similarly, *J*_max_ responses to leaf temperature appeared to be flatter in the Mediterranean genotype at the highest T_growth_ (25 and 30 °C). The rates did not exceed 1.7 at these temperatures, while they reached 2 in the temperate genotype.

### Impact of growth temperature on the limiting step of photosynthesis

**Figure 6.**
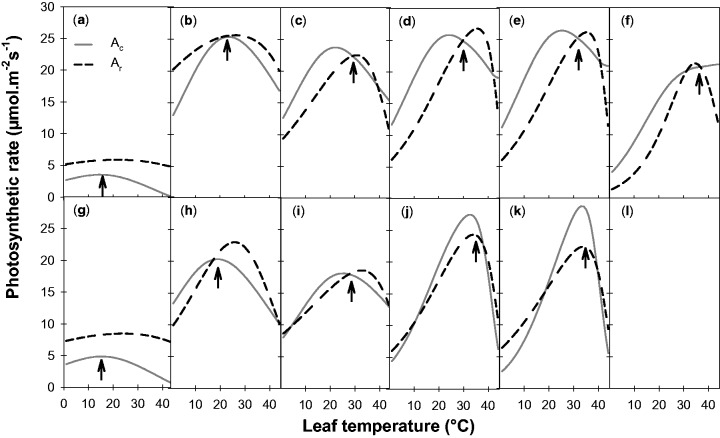
Predicted responses to leaf temperature of the RuBP carboxylation limited (full line, *A*_c_) and the RuBP regeneration limited (dotted line, *A*_r_) assimilation rates at 400 ppm CO_2_ and growth temperatures of 5 °C (a–g), 10 °C (b–h), 20 °C (c–i), 25 °C (d–j), 30 °C (e–k) and 35 °C (f–l). Data are for two alfalfa genotypes of temperate (a–l, G3) and Mediterranean (g–l, 7_7) origins. Arrows indicate the predicted thermal optimum Heat-bleached leaves (l) were unsuitable to derive *V*_cmax_ and *J*_max_ parameters from gas exchange measurements, and therefore no *A*_c_ and *A*_r_ curves were simulated.

The photosynthetic parameters and their responses to leaf temperature **[Supporting Information Table 1]** were used as inputs for the Farquhar model in order to investigate their respective roles in the acclimation of photosynthesis to *T*_growth_. When the whole set of measured parameters was applied, the model proved able to account for the changes in *A*_400_ observed both within (i.e. different *T*_leaf_) and between *T*_growth_ (RMSE < 2 µmol m^−^^2^ s^−^^1^, no significant bias; **[Supporting Information Figure 2]**). In the two genotypes, the relationship between observed and predicted *A*_400_ values did not differ from the 1:1 line (*t*-value < 1.07, *P* > 0.3 for the intercept; *t*-value > 32.0, *P* < 10^−^^3^ for a slope equal to 0.95 ± 0.05). The limiting steps of photosynthesis associated with these simulations are presented in Figure 6. As expected from the response curves of the *V*_cmax_ and *J*_max_ parameters, the temperature dependencies of the assimilation rates limited by RuBP carboxylation (*A*_c_) and RuBP regeneration (*A*_r_) differed at all growth temperatures. In both temperate and Mediterranean genotypes, the optimal temperature predicted by the model (arrows, Fig. 6) increased with rising *T*_growth_ and was closely related to observed *T*_opt_ **[Supporting Information Figure 6]**. Except at 5 °C, *T*_opt_ was determined by the intersection of *A*_400,c_ and *A*_400,r_ in all the situations studied. At *T*_opt_, photosynthesis was thus generally co-limited by *RuBP* carboxylation and *RuBP* regeneration. At 5 °C however, *A*_400,r_ was higher than *A*_400,c_ irrespective of leaf temperature (about 1.5-fold), and *RuBP* carboxylation systematically appeared as the limiting step. Outside the optimal temperature range, the limiting step of photosynthesis also changed as a function of *T*_growth_. Under cold growing conditions (i.e. below 10 °C), *A*_400,c_ fully explained the temperature dependency. In contrast, at warmer *T*_growth_, *A*_400,c_ generally appeared to be the limiting step above *T*_opt_, whereas *A*_400,r_ limited assimilation below *T*_opt_.

Furthermore, a sensitivity analysis was carried out to assess the role of different parameters in acclimation of the *A*_400_ response to temperature. The respective impacts of the plasticity of the *J*_max_^25^*/V*_cmax_^25^ ratio and of changes to the temperature dependencies of *J*_max_ and *V*_cmax_ were examined **[Supporting Information Figure 3]**. Simulations which did not account for modulation of the *J*_max_^25^*/V*_cmax_^25^ ratio (only considering the acclimation of temperature dependencies) showed a much narrower variation in *T*_opt_ between growth temperatures (ranging from 25 to 32 °C) than that actually observed. In contrast, considering changes in the *J*_max_^25^*/V*_cmax_^25^ ratio only (unchanged temperature dependencies) led to an accurate prediction of the acclimation of *T*_opt_ (ranging from 20 to 32 °C), but to more inaccurate simulations of the photosynthetic rates at extreme temperatures (as reflected by the significant increase in RMSE). Under these assumptions, photosynthesis did not decrease as much as expected above 35 °C leaf temperatures for *T*_growth_ such as 25 or 30 °C. Accounting for both processes was necessary to minimize model errors over the broad range of situations studied.

## Discussion

### Acclimation of the thermal optimum of net photosynthesis occurred over a broad range of growing temperatures irrespective of temperate and Mediterranean origins

**Figure 7.**
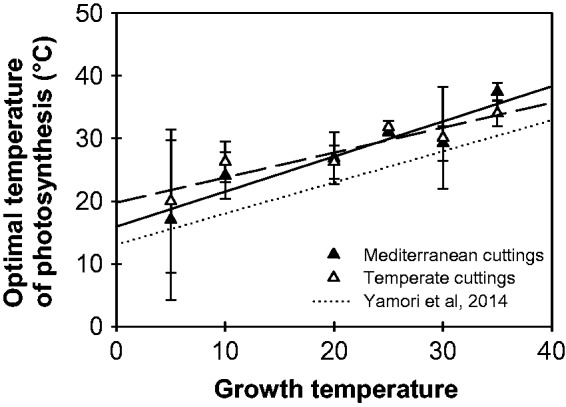
Impact of growth temperature on the optimal temperature of net assimilation rate measured at 400 ppm for two alfalfa genotypes of Mediterranean (filled triangles, 7_7 cutting) and temperate (open triangles, G3 cutting) origins. A linear relationship was fitted for each clone and is represented by a dashed line for Mediterranean and a full line for temperate materials. No significant difference was observed between clones. The regression line found across C_3_ species by Yamori et al. (2014) is also represented (dotted line).

Shifts in the optimal temperature of light saturated photosynthesis (*T*_opt_) as a function of growth temperature have been reported in a number of C_3_ species (e.g. Bunce 2000; Mooney 1980; Yamasaki *et al.* 2002; Yamori *et al.* 2005) and are central to the thermal acclimation of CO_2_ assimilation (Yamori *et al.* 2014). A significant plasticity of *T*_opt_ was also characterized for alfalfa during our experiments, increasing from about 18 °C for leaves grown at 5–32 °C for leaves grown at 35 °C. Acclimation to low and high temperatures were both covered by this range of conditions. Remarkably, the plasticity of *T*_opt_ observed in alfalfa alone matched the range of *T*_opt_ shifts reported by Yamori *et al.* (2014) across a set of contrasting C_3_ species (Fig. 7). The average shift in *T*_opt_ was about 0.48 °C for each 1 °C increase in *T*_growth_ in both temperate and Mediterranean alfalfa cultivars (no significant genotype effect; *y* = 0.48*x + 17.4, *r*^2 ^=^ ^0.85, for the common regression line), as compared with 0.49 °C^−^^1^ on average across C_3_ species (Yamori *et al.* 2014). Whether this high degree of *T*_opt_ acclimation is related to the broad geographic distribution of alfalfa and its perennial growth, or whether it is a more general feature of temperate C_3_ species, still needs to be tested. However, during this study, the same range of *T*_opt_ variations was observed for both of the genotypes studied, irrespective of their origin. No significant difference was found in the *T*_opt_*–T*_growth_ relationship, suggesting that the ability of a genotype to shift *T*_opt_ towards actual growth conditions did not necessarily depend upon the environment in which it was selected. Similarly, Pearcy (1977) and Mooney (1980) found identical *T*_opt_ variations in clones of *Atriplex lentiformis* and *Heliotropum carassivicum* collected from contrasting cool coastal and desert habitats. However, these findings differed from those of several other studies which reported differences in the acclimation potential of populations occupying ecological niches with dissimilar thermal regimes (Berry and Bjorkman 1980; Ishikawa *et al.* 2007).

The reasons for variations in *T*_opt_ as a function of growth temperature had previously been analyzed using the biochemical model developed by Farquhar *et al.* (1980), which assumes that the photosynthetic rate may be limited by either *RuBP* carboxylation by the Rubisco (*A*_c_) or by RuBP regeneration (*A*_r_). Inter-specific differences have been shown in the limiting step of net assimilation at *T*_opt_. Yamori *et al.* (2010) suggested that C_3_ plant species could belong to one of three categories: net assimilation limited at *T*_opt_ by *A*_c_ (i), by *A*_r_ (ii) or co-limited and determined by the intersection of *A*_c_ and *A*_r_ (iii). Our results showed that alfalfa belonged to the co-limitation group in all cases, except at 5 °C *T*_growth_ (*A*_c_ group). This behaviour was consistent with other cold tolerant temperate species such as wheat, rye or faba bean (Yamori *et al.* 2010). In the case where *T*_opt_ is determined by the co-limitation of *A*_c_ and *A*_r_, the optimum temperature of net assimilation can shift through changes in the temperature dependencies of each partial reaction, and also simply because of changes to the balance between *A*_c_ and *A*_r_, even if their temperature response does not change (Farquhar and Von Caemmerer 1982). In our study, both contributed to the observed shifts in alfalfa. The temperature dependencies of *V*_cmax_ and *J*_max_ were modified in the two genotypes studied as a function of growth temperature, resulting in shifts of *A*_c_ and *A*_r_. Similarly, the *J*_max_^25^*/V*_cmax_^25^ ratio decreased regularly in leaves grown at between 5 and 35 °C. However, sensitivity analysis revealed that the *J*_max_^25^*/V*_cmax_^25^ ratio had the strongest impact in determining *T*_opt_ **[Supporting Information Figure 3]**. Changes to the *J*_max_^25^*/V*_cmax_^25^ ratio have also been found to be the variable which correlates best to shifts in *T*_opt_ among different species (Onoda *et al.* 2005; Yamori *et al.* 2010).

The increase in this ratio at low temperatures had previously been associated with elevated concentrations of enzymes involved in the capacity of the Calvin cycle and thylakoid reactions to regenerate *RuBP* (Antolin *et al.* 2005; Berry and Bjorkman 1980) or decreased investment in Calvin Cycle capacity. For instance, Ishikawa *et al.* (2007) reported an increased ratio between fructose-1,6-biphosphatase and Rubisco contents in *Plantago asiatica* genotypes able to adapt their *T*_opt_ at low temperatures. In our study, the *J*_max_^25^*/V*_cmax_^25^ ratio in the two genotypes displayed the same response to temperature. A significant plasticity of nitrogen partitioning in the photosynthetic apparatus was thus observed irrespective of the origin of genotypes, which might explain why the same range of *T*_opt_ variations was observed.

### Homeostasis of light-saturated photosynthesis occurred within a narrower range of growing temperatures

Changes to *T*_growth_ not only modified nitrogen partitioning in the photosynthetic apparatus but also dramatically altered leaf growth, leaf structure (as reflected by SLA) and the leaf nitrogen content. Despite these growth modifications, an apparent homeostasis of light saturated net photosynthesis (*A*_400_^25^) was observed within a broad range of temperatures from 10 to 30 °C, irrespective of the origin of the cultivars. This temperature homeostasis of photosynthesis may play an important role in the ability of alfalfa to grow successfully in habitats with contrasting temperature regimes, and to adapt to changes in temperatures over its extended growing season (Berry and Bjorkman 1980). Yamori *et al.* (2014) indeed indicated that perennial herbaceous species generally display greater temperature homeostasis of photosynthesis than deciduous woody and annual herbaceous species.

The calculation of photosynthetic nitrogen use efficiency (PNUE) at each growth temperature suggested that these growth modifications could be adaptive. Indeed, PNUE values peaked at intermediate temperatures (25 °C), falling steadily on either side of the curve **[Supporting Information Figure 4]**. The photosynthetic capacity per unit of leaf nitrogen was thus not constant and was reduced under high and low growth temperatures. Modifications to CO_2_ diffusion, nitrogen allocation between photosynthetic and non-photosynthetic nitrogenous compounds, and the kinetics of photosynthetic enzymes could be three possible reasons for these reductions in *PNUE* (Field and Mooney 1986; Hikosaka 2004; Reich *et al.* 1998). By modifying the structure of leaves, and increasing their nitrogen content at high and low growth temperatures, alfalfa developed responses which contributed to maintaining high rates of net assimilation per unit of leaf area. However, at the most extreme temperatures (5 and 35 °C), these structural acclimations were not enough to compensate for the important drop in PNUE values, causing significant reductions in *A*_400_^25^. The same decrease was seen to affect *A*_400_^Opt^ (Fig. 3) **[Supporting Information Figure 4]**. This suggests that, at least in alfalfa, adaptive acclimation may occur within a much narrower range of growth temperatures (10–30 °C) than that within which *T*_opt_ may shift (5–35 °C in this study).

Thermal stresses irreversibly disrupting the integrity of the photosynthetic apparatus most likely reduced maximal photosynthesis at the two extreme growth temperatures (Berry and Bjorkman 1980). Although not characterized, the reduction in net assimilation seen below 10 °C might be related to photo-inhibition (Jones and Kok 1966a,b; Louarn et al. 2008). This hypothesis is consistent with the high *J*_max_^25^*/V*_cmax_^25^ ratio observed at these temperatures, where *A*_c_ was shown to be the limiting factor of CO_2_ assimilation. Over-investment in the electron transport chain may indeed provide protection against photo-oxidative damage (Hikosaka 2005). No difference in response was found between the two genotypes with respect to this stress. On the other hand, at high growth temperatures, a drop in the net assimilation rate was seen in both genotypes, but was dramatically more pronounced in the Mediterranean cuttings. Symptoms of thermal stress were apparent in the form of heat-bleached leaves in the Mediterranean material **[Supporting Information Figure 1]** (Feierabend and Mikus 1977; Smillie *et al.* 1978). The stress only affected new leaves that emerged after the transfer at 35 °C, and resulted in leaves lacking chlorophylls (as assessed by chlorophyll chromatography, not shown). All the 7_7 cuttings and about 30% of the plants in the Barmed cultivar were affected. McKenzie *et al.* (1988) had previously noticed that a genetic variability existed in alfalfa concerning heat-induced reductions in maximal photosynthesis.

### Temperate and Mediterranean genotypes differed in their tolerance to high temperatures

Except at the stressful growth temperature of 35 °C, the temperate and Mediterranean genotypes did not differ in terms of their *A*_400_^25^ or *A*_400_^Opt^. However, at high leaf temperatures (42 °C), the photosynthetic rate was almost always lower in the Mediterranean genotype (10, 25 and 30 °C *T*_growth_). Leaves showing no apparent signs of thermal stress, and with similar maximal photosynthesis, thus displayed different temperature responses in terms of net photosynthesis. These differences were linked to those in the acclimation of temperature dependencies of the photosynthetic parameters *V*_cmax_ and *J*_max_ as a function of *T*_growth_ (Fig. 5). They were modified in both genotypes, but these changes differed in nature. In the temperate genotype, *V*_cmax_ conserved a typical, rising Arrhenius response curve irrespective of growth temperature, while responses decreasing at high leaf temperatures were observed in the Mediterranean genotype. Such decreases in *V*_cmax_ at high leaf temperatures had previously been reported in several species (Sage *et al.* 2008; Yamori *et al.* 2006 , 2012). They were associated with reversible changes in the activation state of the Rubisco induced by high leaf temperatures, with gradual de-activation occurring under moderate heat stresses (Salvucci and Crafts-Brandner 2004; Yamori *et al.* 2012). This *V*_cmax_ reduction resulted primarily from a reduction in the activity of Rubisco activase (Kumar *et al.* 2009; Kurek *et al.* 2007; Sage *et al.* 2008), rather than from a reduction of Rubisco catalytic activity (Galmés *et al.* 2014, 2015). The difference between the two genotypes might also have resulted from their varying abilities to produce heat-shock proteins, affording better protection of the various components of the photosynthetic apparatus in temperate genotype (Burke 2001). Overall, these biochemical responses resulted in net assimilation rates that were more limited at high leaf temperatures in the Mediterranean genotype, because of depreciated *A*_c_ values.

Given the thermal differences in their original habitats, the greater sensitivity of the Mediterranean material to high temperatures had not necessarily been anticipated. Whether this genetic variability was circumstantial (sampling effect), or whether it had some ecological significance and was indicative of adaptation to local conditions, now needs to be confirmed. However, the evidence of a greater sensitivity of Mediterranean plants was consistent in the two types of plant material tested (seedlings from the Barmed cv. and a clone selected from the Demnate cv.). Observations in the same genotype of a greater thermal sensitivity of the Rubisco in response to short-term heat exposure, and of more severe damage in response to prolonged heat stress (heat bleaching), were also coherent with a niche differentiation. One possible explanation for this counter-intuitive result is that many species from the Mediterranean basin, even herbaceous perennials, actually develop stress avoidance rather than stress tolerance strategies, and are dormant or non-productive during periods of high temperatures in the summer (Volaire *et al.* 2009, 2014). Typical Mediterranean plants are thus adapted to growing during the cooler seasons rather than to maintaining active photosynthesis during heat waves (MacColl and Cooper 1967).

## Conclusions

To conclude, a significant intra-specific variability was found in terms of the thermal acclimation of photosynthesis in alfalfa. Within the non-stressful range of growth temperatures, this was ascribed to changes in the temperature dependence of the maximum rate of Rubisco carboxylation rather than to differences in nitrogen partitioning within the photosynthetic apparatus, as reported previously (Hikosaka 2005; Ishikawa *et al.* 2007). This resulted in differentiated responses of net photosynthesis at high leaf temperatures, but not in different maximal photosynthetic rates, rates at 25 °C or shifts of optimal temperature. The response of the Mediterranean genotypes tested was consistent with a strategy of thermal stress avoidance, so they do not seem suitable to be used as a genetic resource for breeding to improve the thermal tolerance of photosynthesis. On the other hand, room for improvement exists to increase the summer photosynthesis of southern varieties.

## Sources of Funding

This study received support from the CLIMAGIE project of INRA’s ACCAF metaprogramme, the REFORMA project funded by the ARIMNet call of the ERA-CAPS 7th EU Framework Programme and the MODEXTREME project funded by the FP7. Serge Zaka’s PhD grant was financed by the Poitou-Charentes Regional Council (CPER).

## Contributions by the Authors

S.Z., G.L. and E.F. designed the experiments and conducted measurements. S.Z. and G.L. contributed to model development and ran simulations. S.Z. performed data analyses. B.J. and G.L rose funding for these research. All of the authors contributed to writing the manuscript.

## Conflict of Interest Statement

None declared.

## Supplementary Material

Supplementary Data

## Supporting Information

The following additional information is available in the online version of this article —

**Sup. Fig. 1.** Pictures of alfalfa leaves of Mediterranean and temperate cultivars grown at different temperatures.

**Sup. Fig. 2.** Comparison of measured and simulated net assimilation rates for alfalfa leaves from the two temperate and Mediterranean cuttings studied.

**Sup. Fig. 3.** Predicted responses to leaf temperature of the RuBP carboxylation limited and the RuBP regeneration limited assimilation rates at growth temperatures ranging from 5°C to 35°C under three scenarios: i) using photosynthetic parameters actually measured ii) assuming a constant J_max_^25^/ V_cmax_^25^ ratio across growth temperatures or iii) assuming unchanged temperature dependencies across growth temperatures.

**Sup. Fig. 4.** Impact of growth temperature on the Photosynthetic Nitrogen Use Efficiency (PNUE) of alfalfa leaves on two genotypes from Mediterranean and temperate origins.

**Sup. Fig. 5.** Impact of growth temperature on the maximal light-saturated photosynthesis at optimal temperature for two genotypes from Mediterranean and temperate origins.

**Sup. Fig. 6.** Relationship between observed and predicted optimal temperatures.

**Sup. Table 1.** Parameters for temperature dependencies of the maximum rate of Rubisco carboxylation (V_cmax_) and the maximum electron transport rate (J_max_) for each growth temperature.

## Appendix 1

**Table A1.** Abbreviations of all the variables, parameters and symbols used

*A*	µmol CO_2_ m^−2^ s^−1^	Net photosynthetic rate
*A*_400_	µmol CO_2_ m^−2^ s^−1^	Net photosynthetic rate at 400 ppm CO_2_
*A*_400_^25^	µmol CO_2_ m^−2^ s^−1^	Net photosynthetic rate at 400 ppm CO_2_ and 25 °C leaf temperature
*A*_400_^growth^	µmol CO_2_ m^−2^ s^−1^	Net photosynthetic rate at 400 ppm CO_2_ and leaf at the growth temperature
*A*_400_^opt^	µmol CO_2_ m^−2^ s^−1^	Net photosynthetic rate at 400 ppm CO_2_ and thermal optimum
*A*_c_	µmol CO_2_ m^−2^ s^−1^	Rubisco-limited rate of carboxylation
*A*_r_	µmol CO_2_ m^−2^ s^−1^	Electron transport-limited rate of carboxylation
*C*_a_	ppm	Ambient CO_2_ partial pressure
*C*_i_	ppm	Intercellular CO_2_ partial pressure
*ΔH*_a_	J mol^−1^	Enthalpy of activation
*ΔH*_d_	J mol^−1^	Enthalpy of de-activation
*J*_max_	µmol m^−2^ s^−1^	Maximum electron transport rate
*K*_c_	µmol mol^−1^	Michaelis-Menten constant for CO_2_
*K*_o_	mmol mol^−1^	Michaelis-Menten constant for O_2_
*L*_area_	cm^2^	Final leaf area
*N*_a_	g m^−2^	Amount of nitrogen per unit leaf area
*O*	KPa	Oxygen partial pressure
*P*^25^	µmol m^−2^ s^−1^	Value of V_cmax_ or J_max_ at 25 °C
PNUE	µmol CO_2_ g N^−1^ s^−1^	Photosynthetic nitrogen use efficiency
PPFD	µmol photon m^−2^ s^−1^	Photosynthetic photon flux density
*Γ**	µmol mol^−1^	CO_2_ compensation point in the absence of dark respiration
*R*	KJ mol^−1^ K^−1^	Universal gas constant for perfect gases
*R*_d_	µmol m^−2^ s^−1^	Mitochondrial respiration
*RuBP*	–	Ribulose-1,5-biphosphate
*ΔS*	J mol^−1^ K^−1^	Entropy term
SLA	cm^2^ g	Specific leaf area
*T*_growth_	°C	Growth temperature
*T*_leaf_	°C	Leaf temperature
*T*_max_	°C	Maximum temperature of a process
*T*_min_	°C	Minimum temperature of a process
*T*_opt_	°C	Thermal optimum of photosynthesis
*V*_cmax_	µmol m^−2^ s^−1^	Maximum rate of Rubisco carboxylation
VPD	kPa	Vapour pressure deficit
