# Supplementary material for: Intraspecific variation in thermal acclimation of photosynthesis across a range of temperatures in a perennial crop
Source: AoB Plants. 2016 Jul 11;8:plw035. doi: 10.1093/aobpla/plw035 (PMC4940478; doi:10.1093/aobpla/plw035)
Supplement: Supplementary Data [file supp_8_plw035_index.html]

Intraspecific variation in thermal acclimation of photosynthesis across a range of temperatures in a perennial crop — Supplementary Data 

# Intraspecific variation in thermal acclimation of photosynthesis across a range of temperatures in a perennial crop

## Supplementary Data

files

- Supplementary Data - pdf file
- Supplementary Data - doc file
