# Supplementary material for: Intraspecific variation in thermal acclimation of photosynthesis across a range of temperatures in a perennial crop
Source: AoB Plants. 2016 Jul 11;8:plw035. doi: 10.1093/aobpla/plw035 (PMC4940478; doi:10.1093/aobpla/plw035)
Supplement: Supplementary Data [file supp_plw035_aobplants-15263-T-s03.pdf]

[Research Article]

Intraspecific variation in thermal acclimation of photosynthesis across a range of temperatures in a perennial crop.

Serge Zaka, Ela Frak, Bernadette Julier, François Gastal, Gaëtan Louarn*

INRA UR4 URP3F, BP6, F86600 Lusignan, France

*corresponding author: gaetan.louarn@lusignan.inra.fr

Tel: 33 (0)5 49 55 60 63

Fax : 33 (0)5 49 55 60 68

Running title: Thermal acclimation of photosynthesis in alfalfa
