# Supplementary material for: Intraspecific variation in thermal acclimation of photosynthesis across a range of temperatures in a perennial crop
Source: AoB Plants. 2016 Jul 11;8:plw035. doi: 10.1093/aobpla/plw035 (PMC4940478; doi:10.1093/aobpla/plw035)
Supplement: Supplementary Data [file supp_plw035_aobplants-15263-T-s02.doc]

**Supplementary information**

| **5°C**  1 cm | **10°C** | **15°C** | **20°C** | **25°C** | **30°C** | **35°C** |
| --- | --- | --- | --- | --- | --- | --- |
| 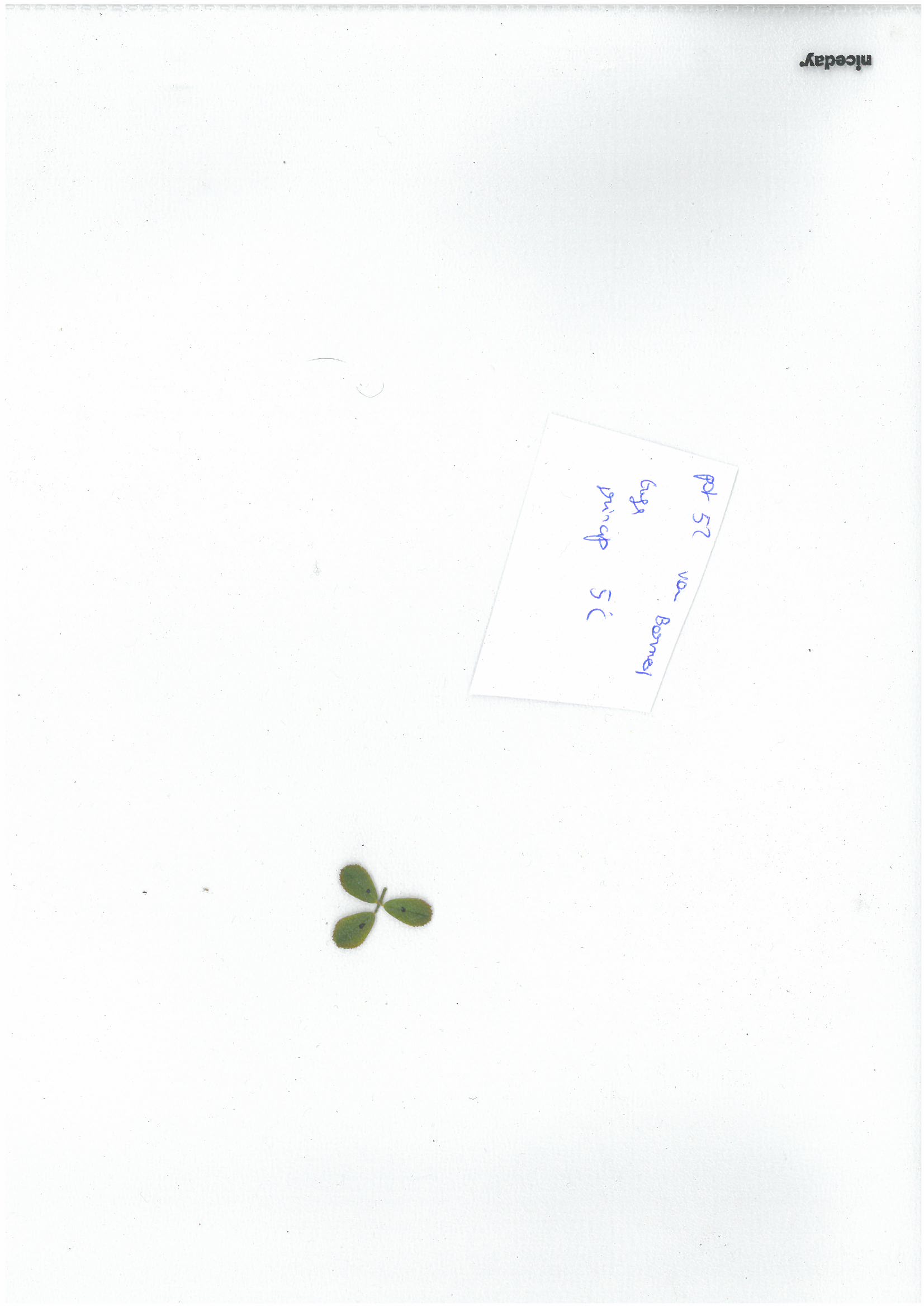 | 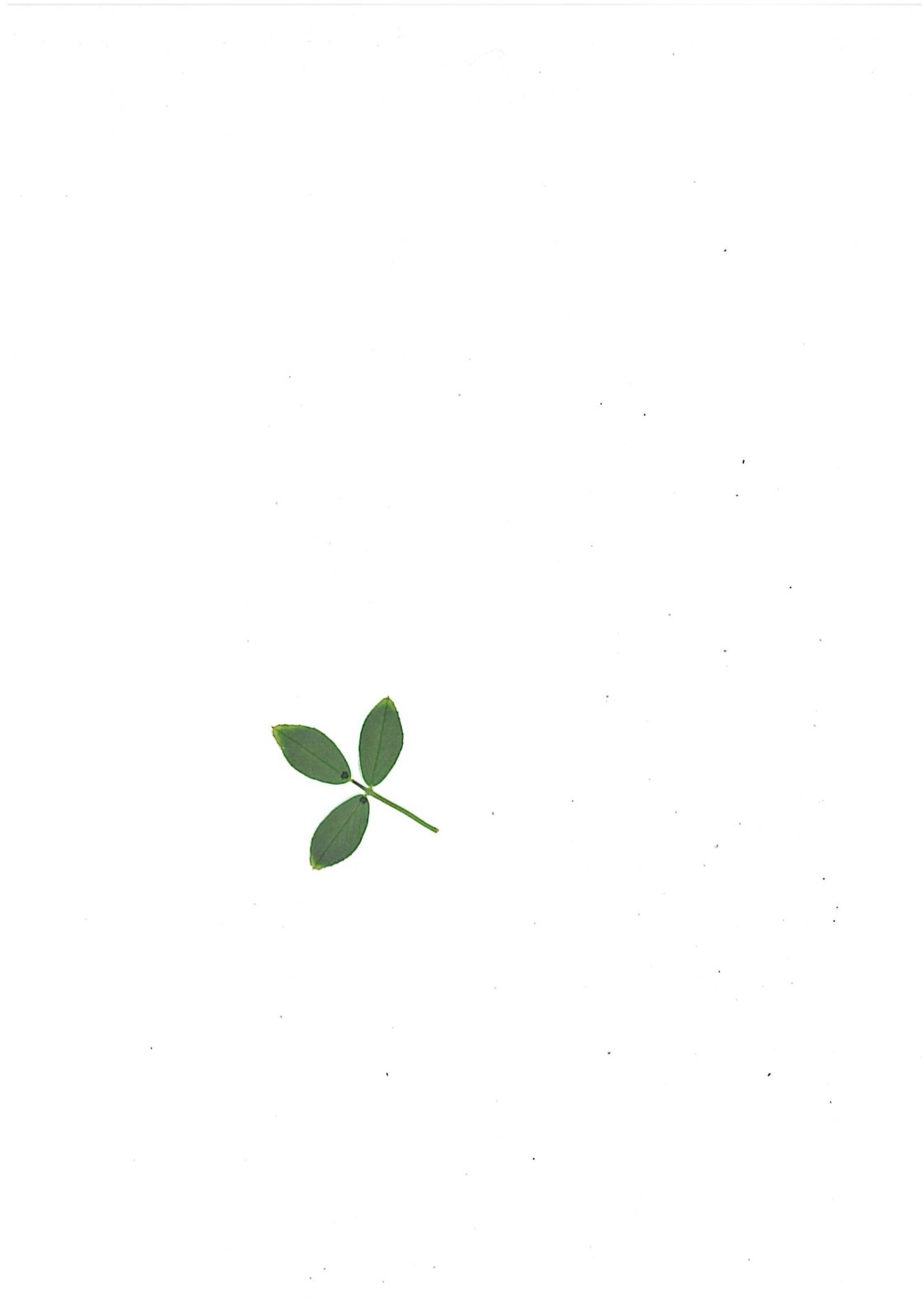 | 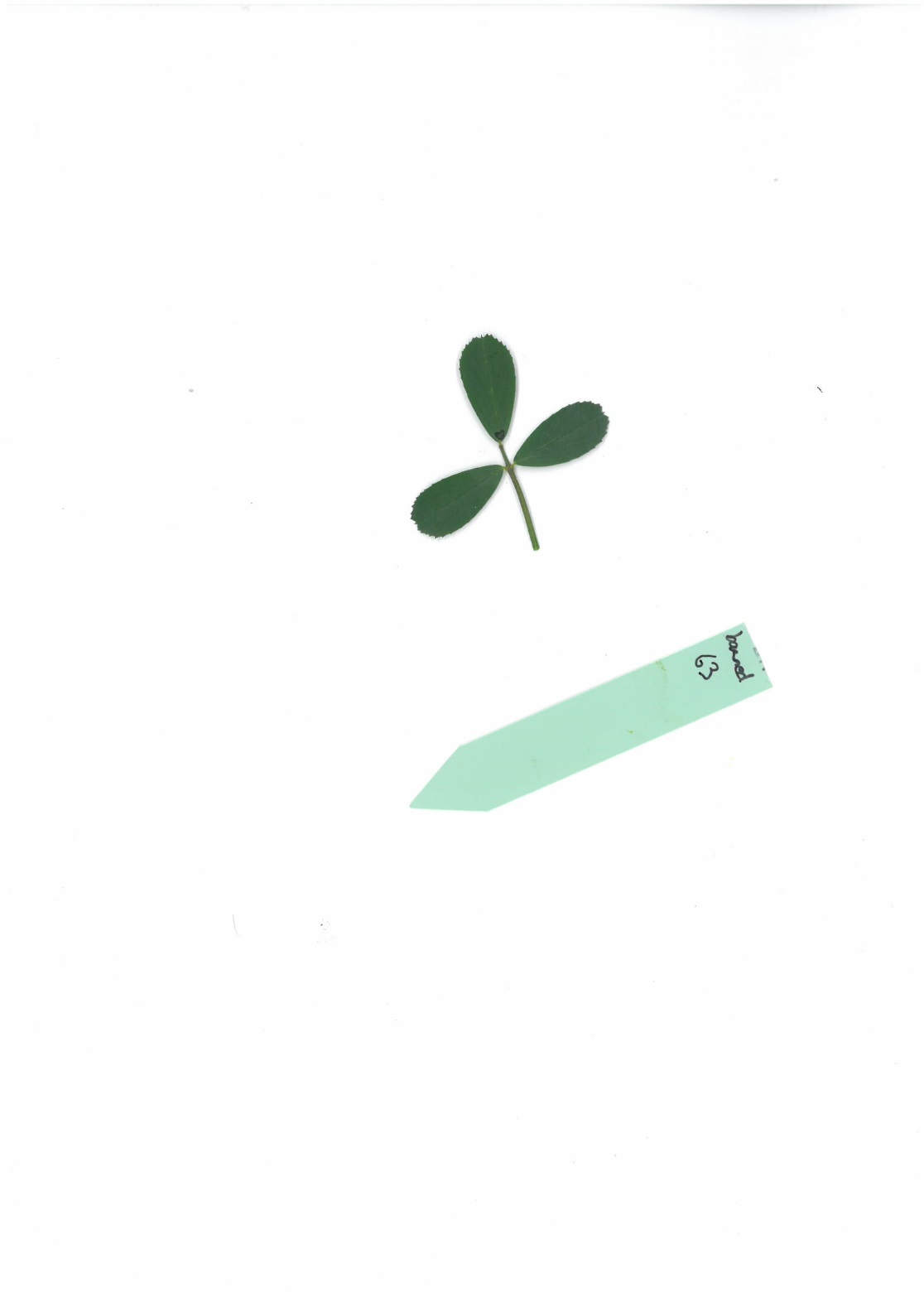 | 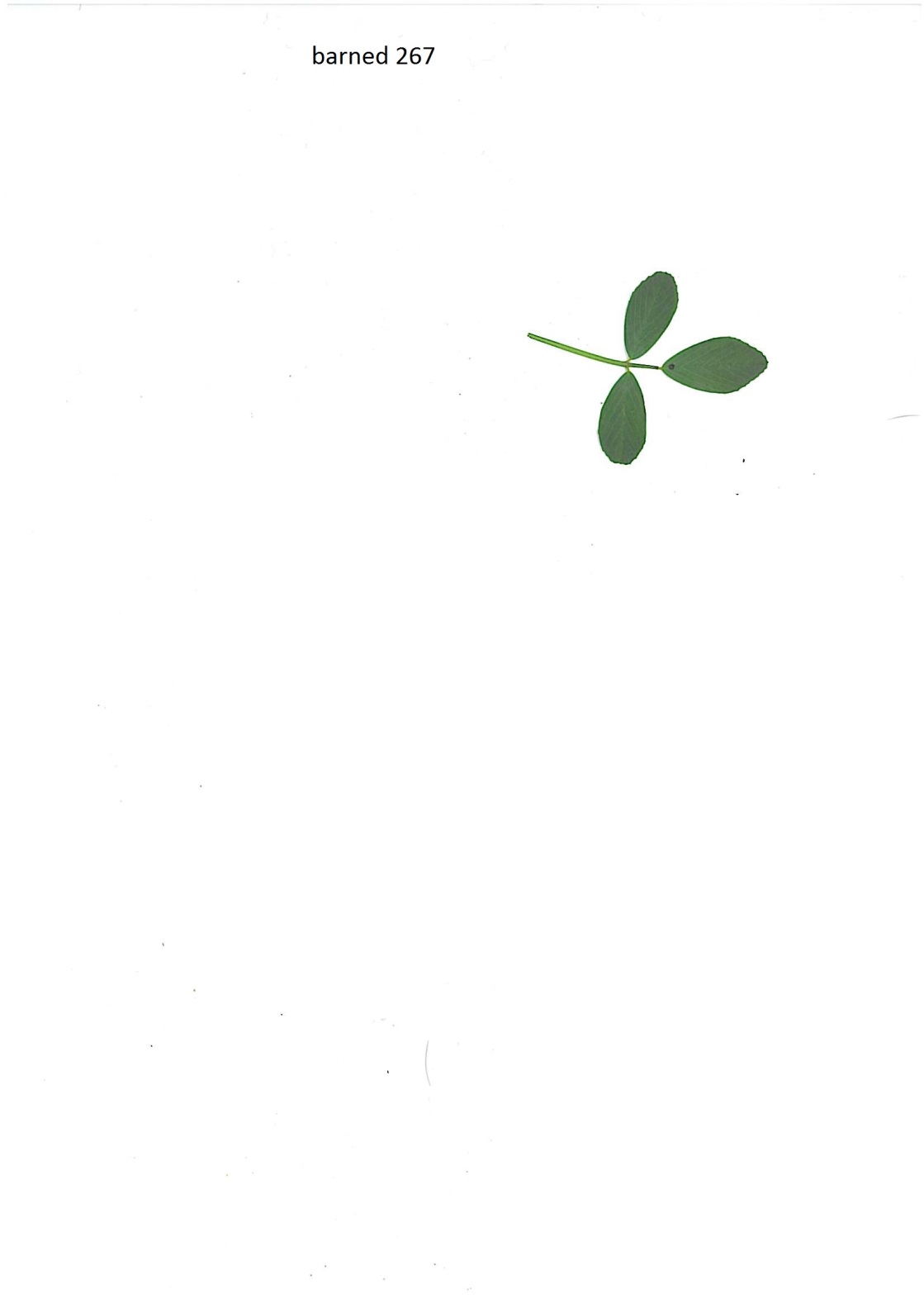 | **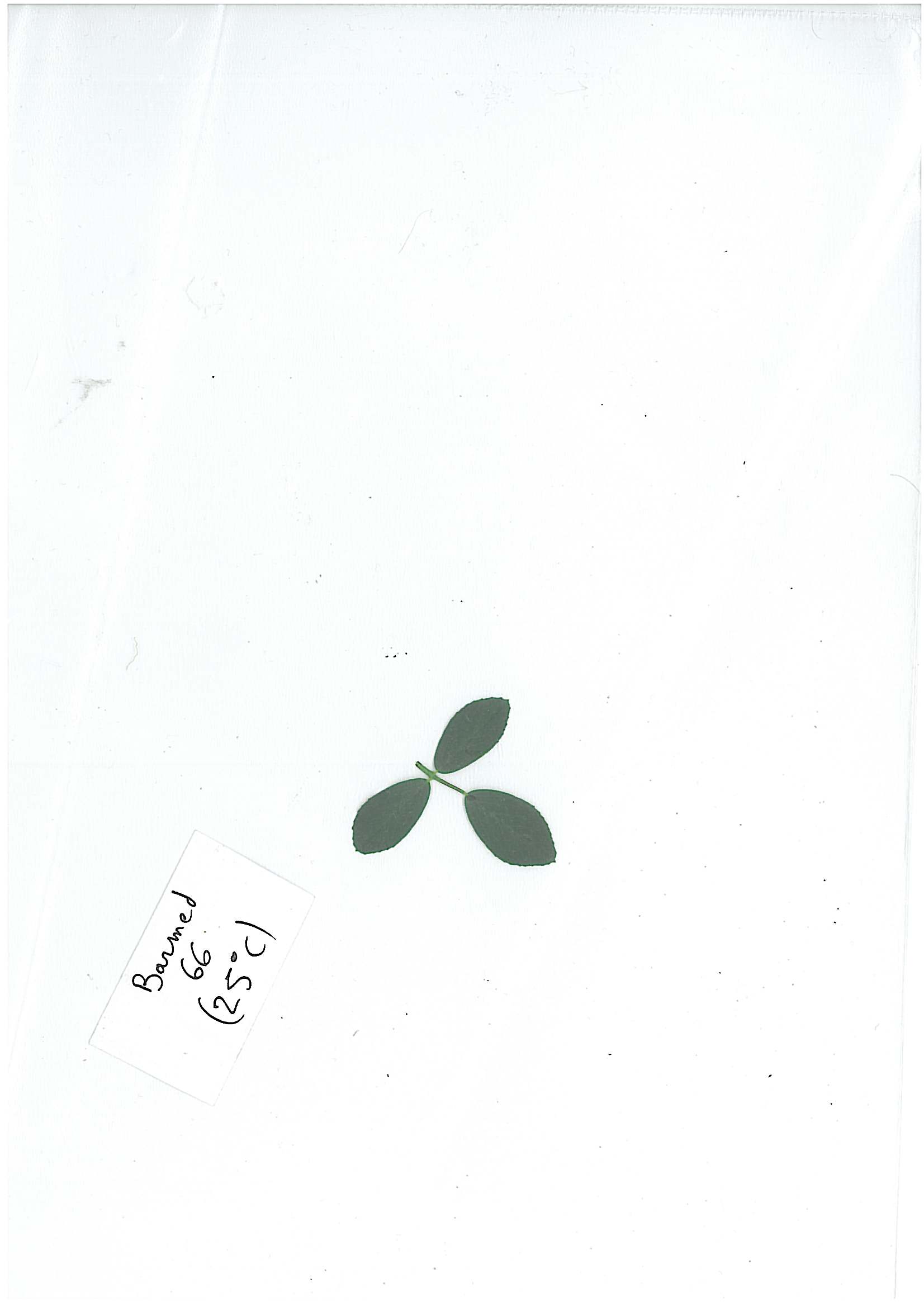** | **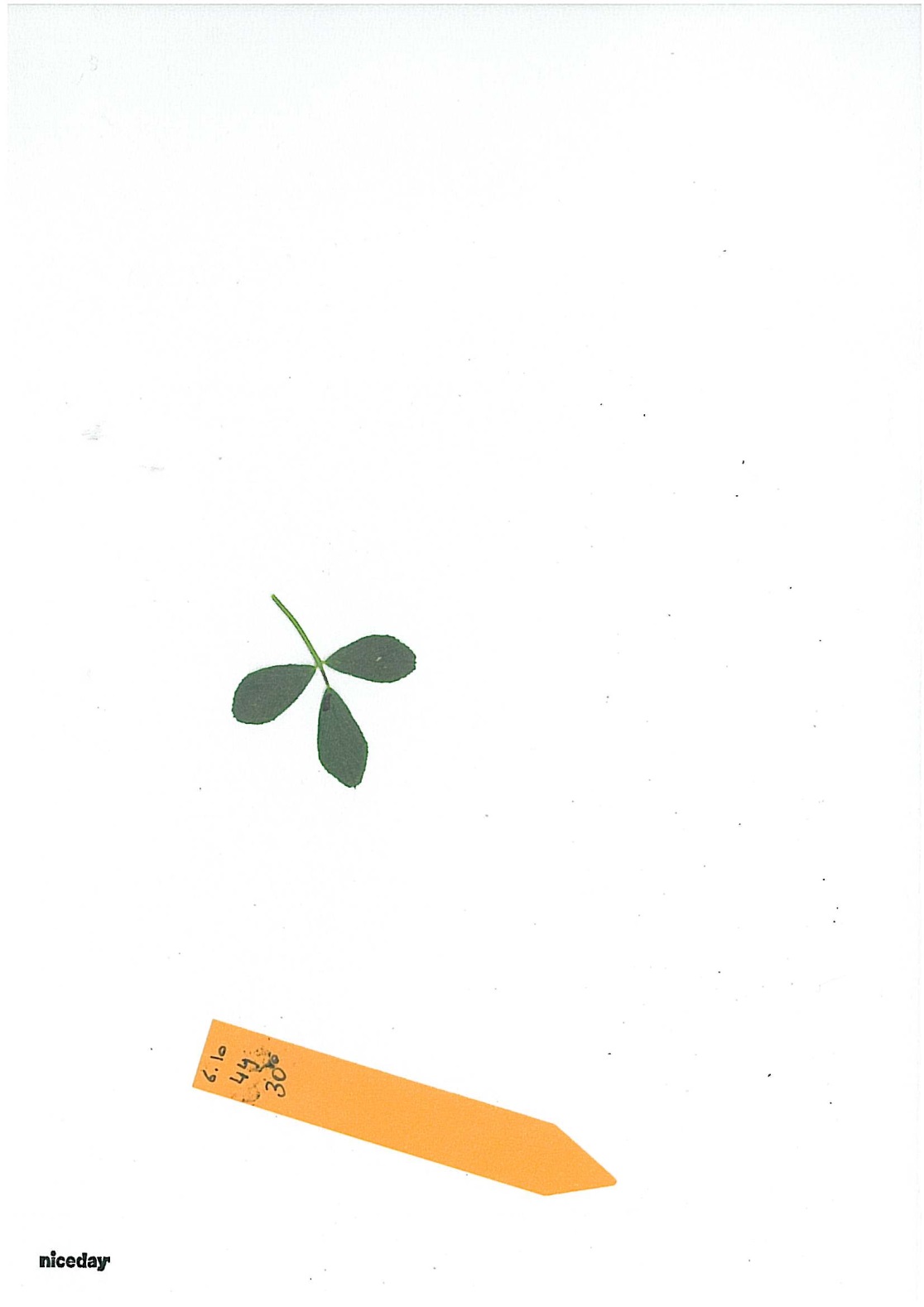** | **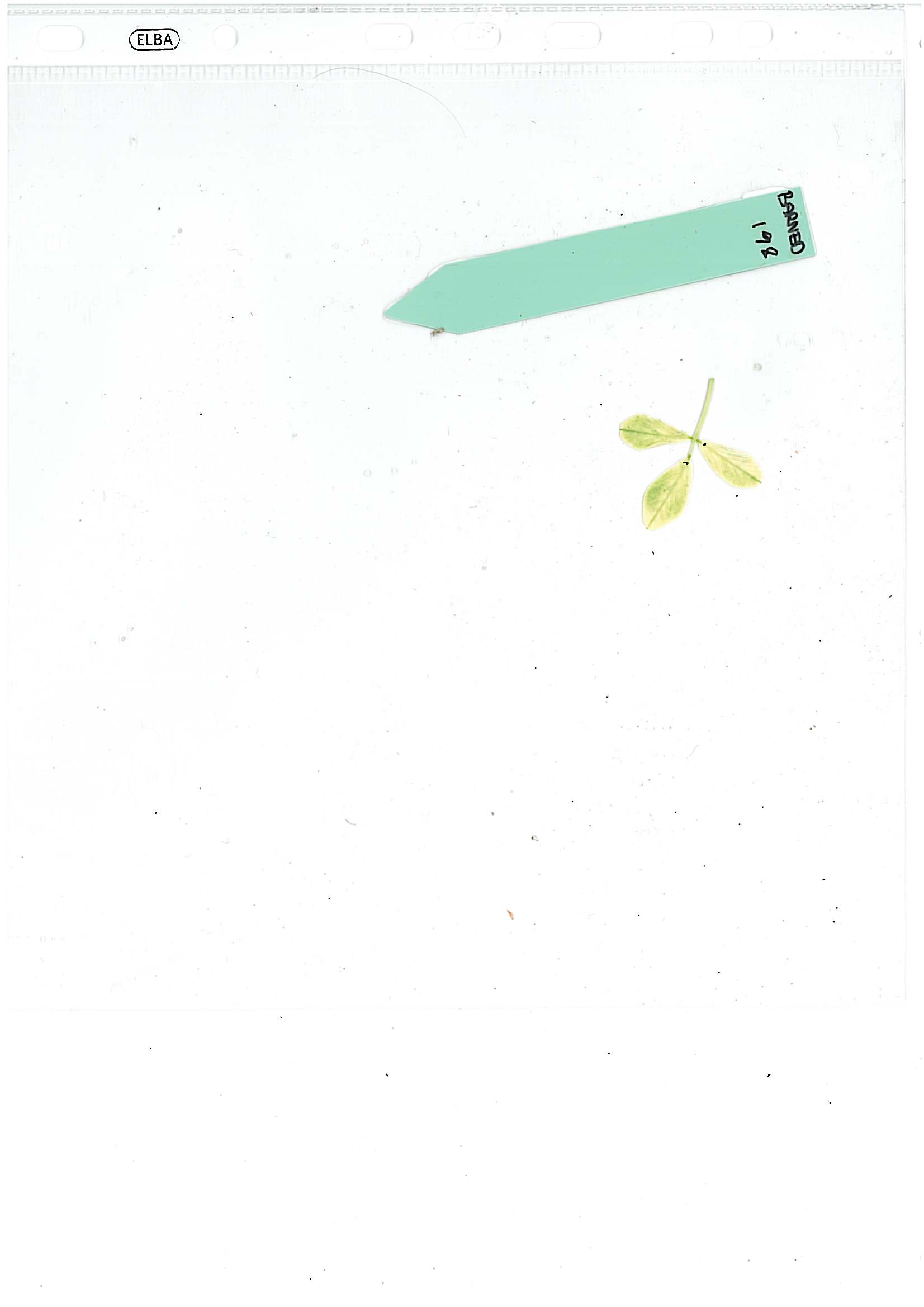** |
| 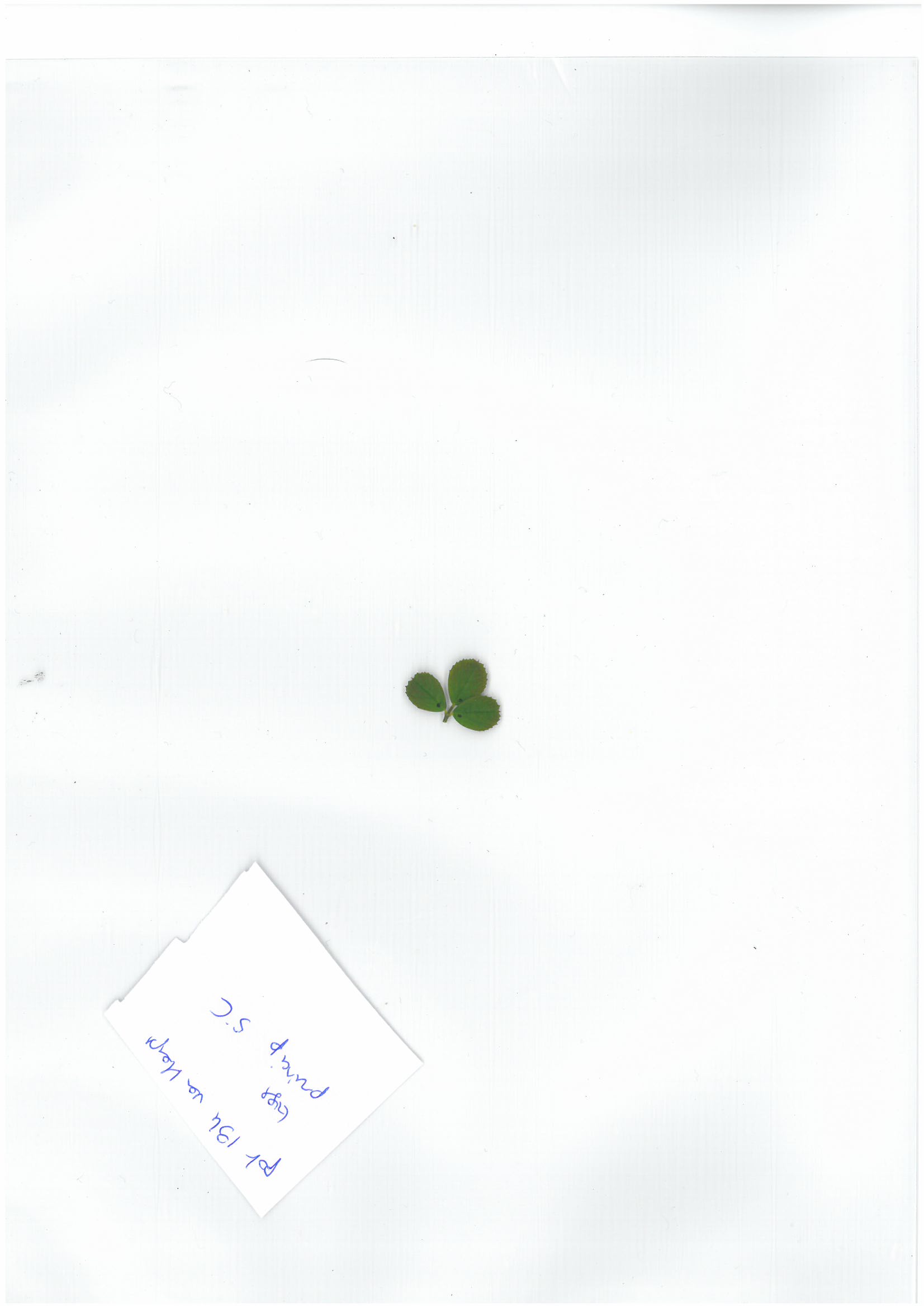 | 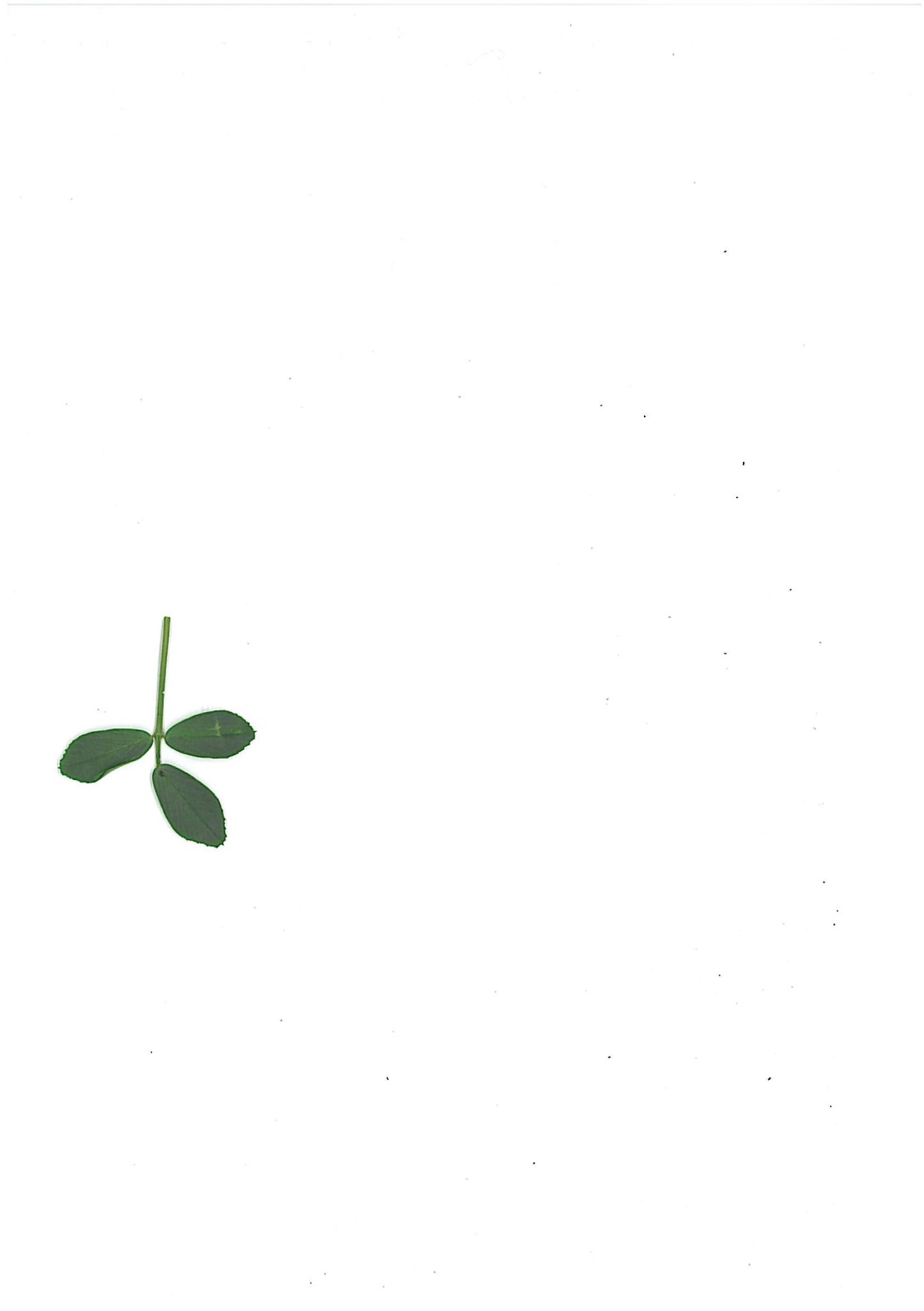 | 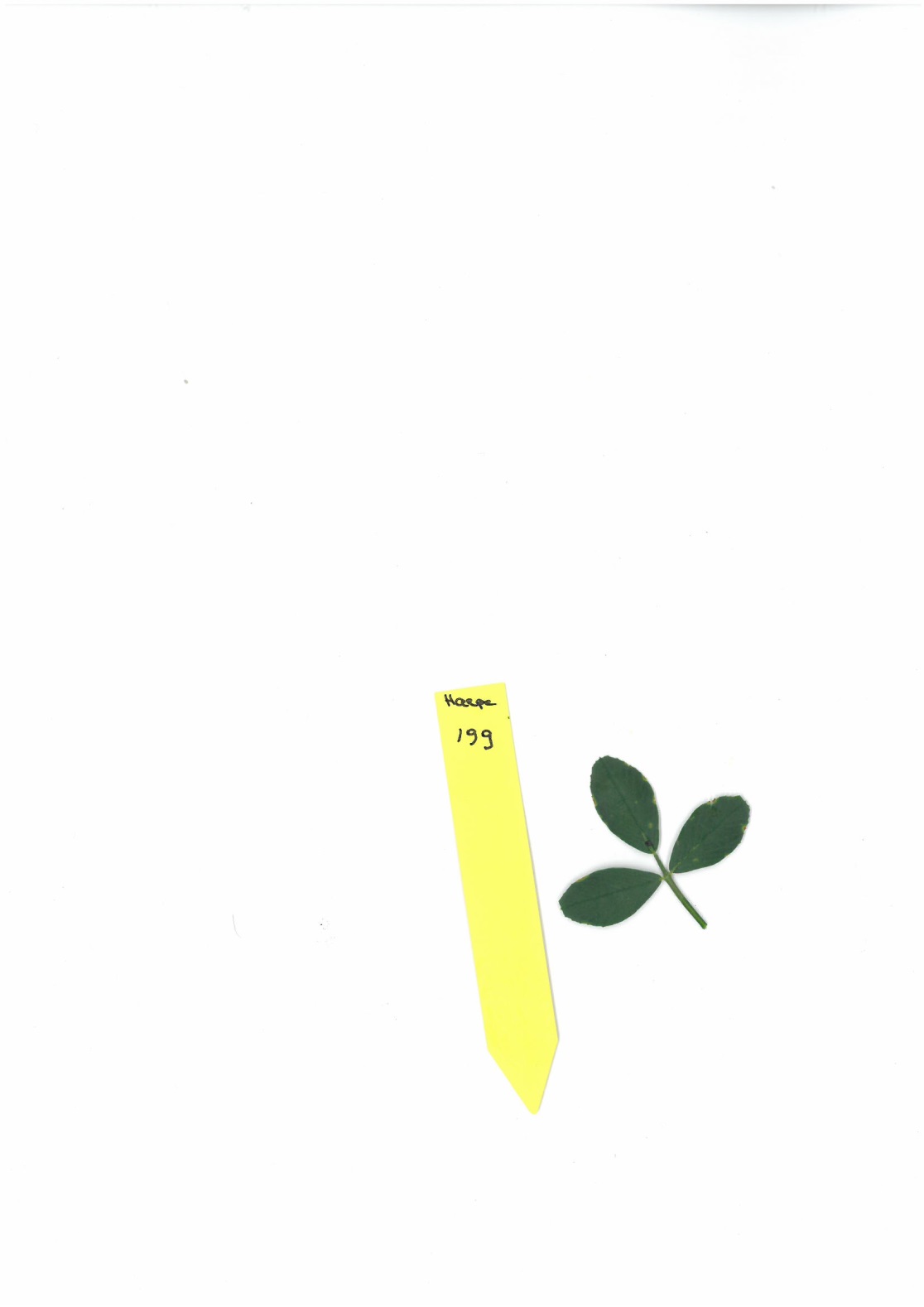 | **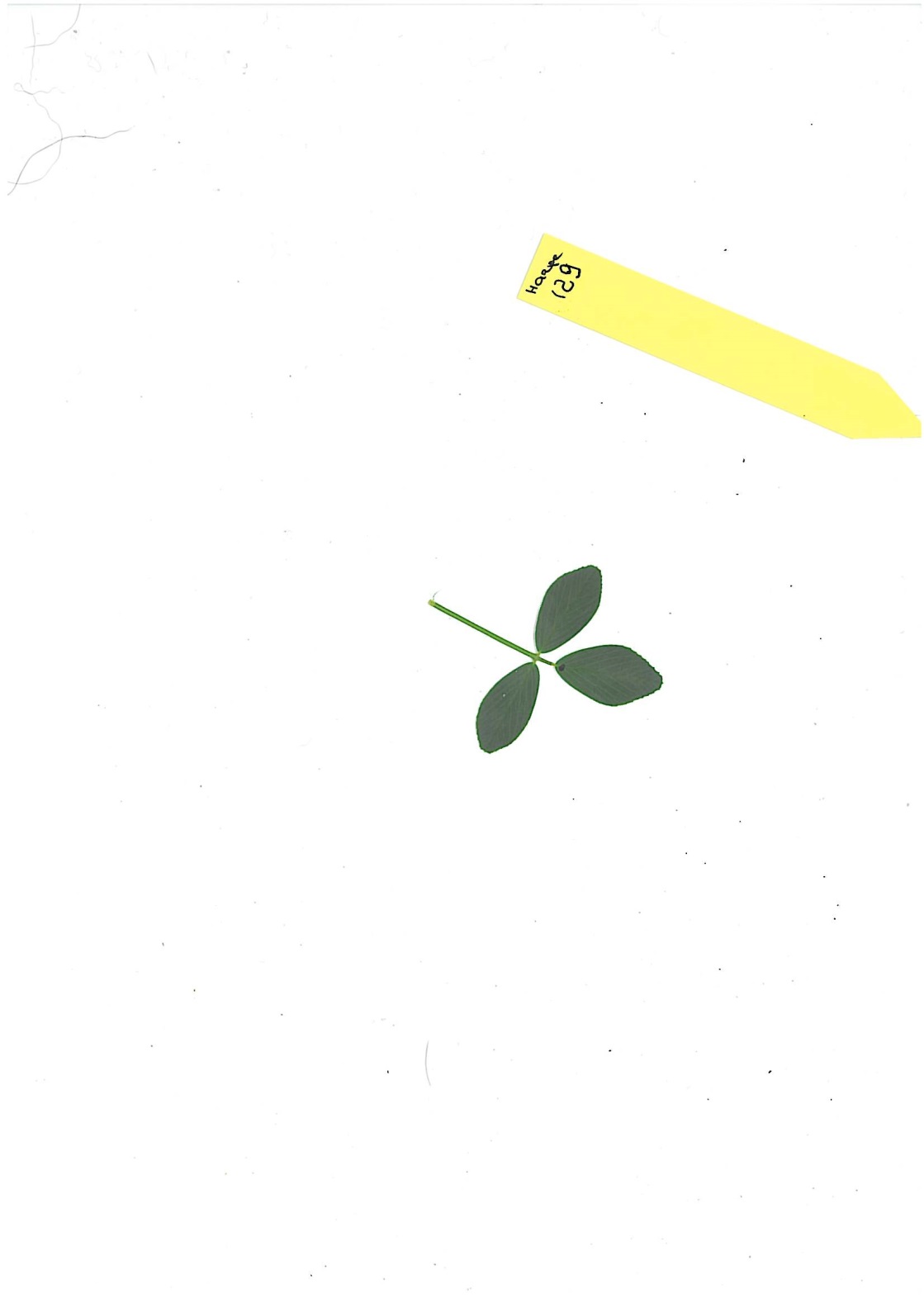** | **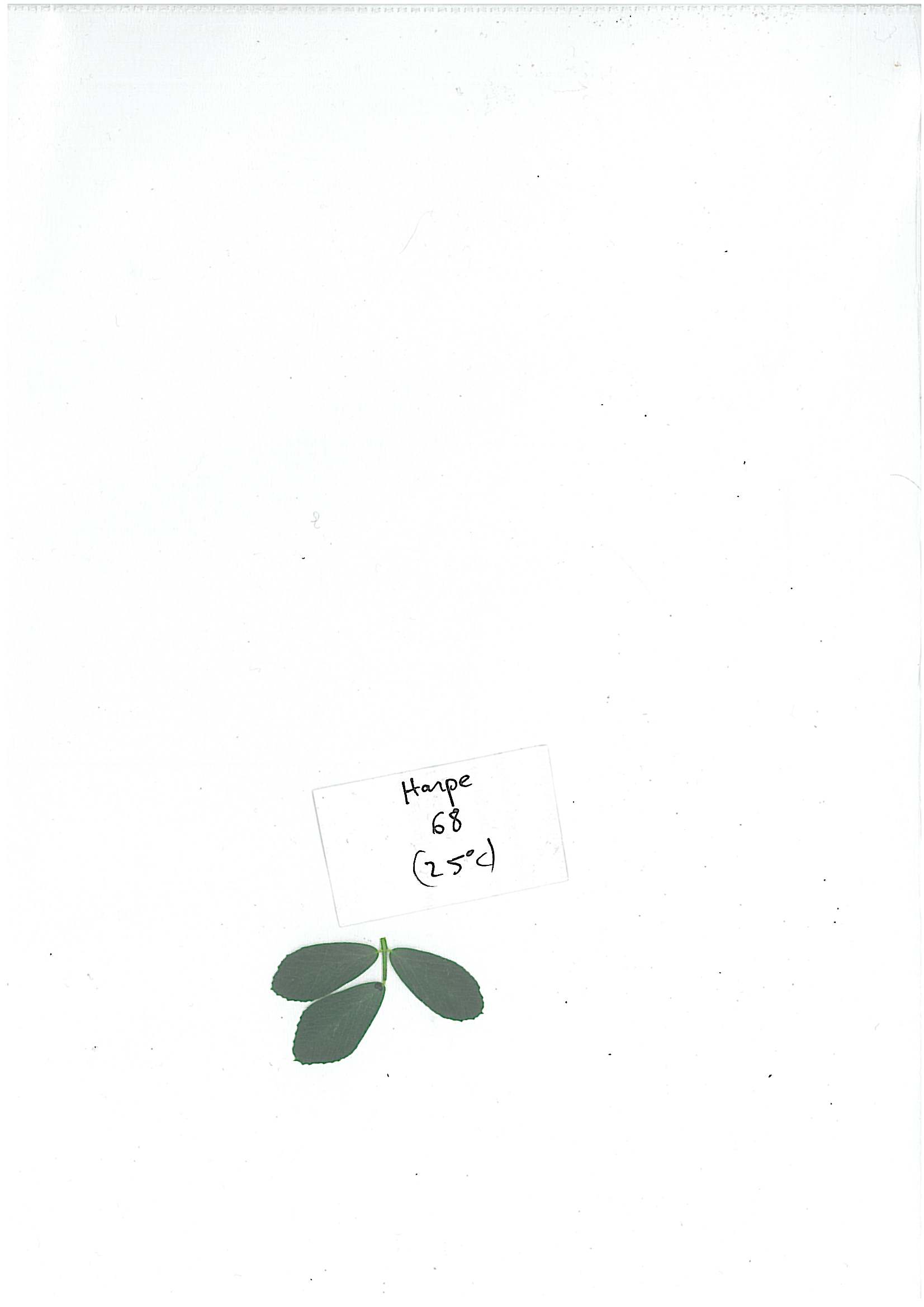** | **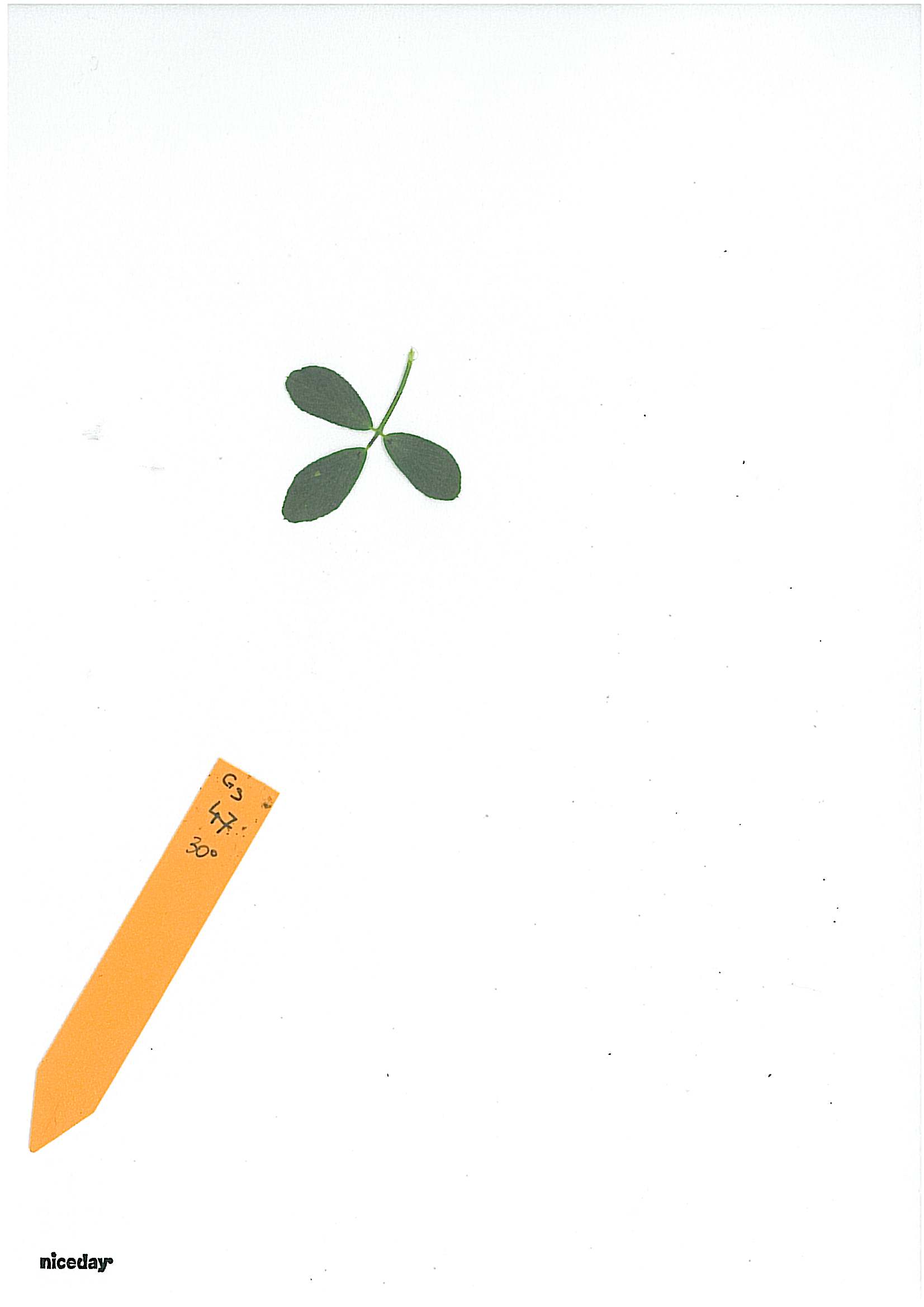** | **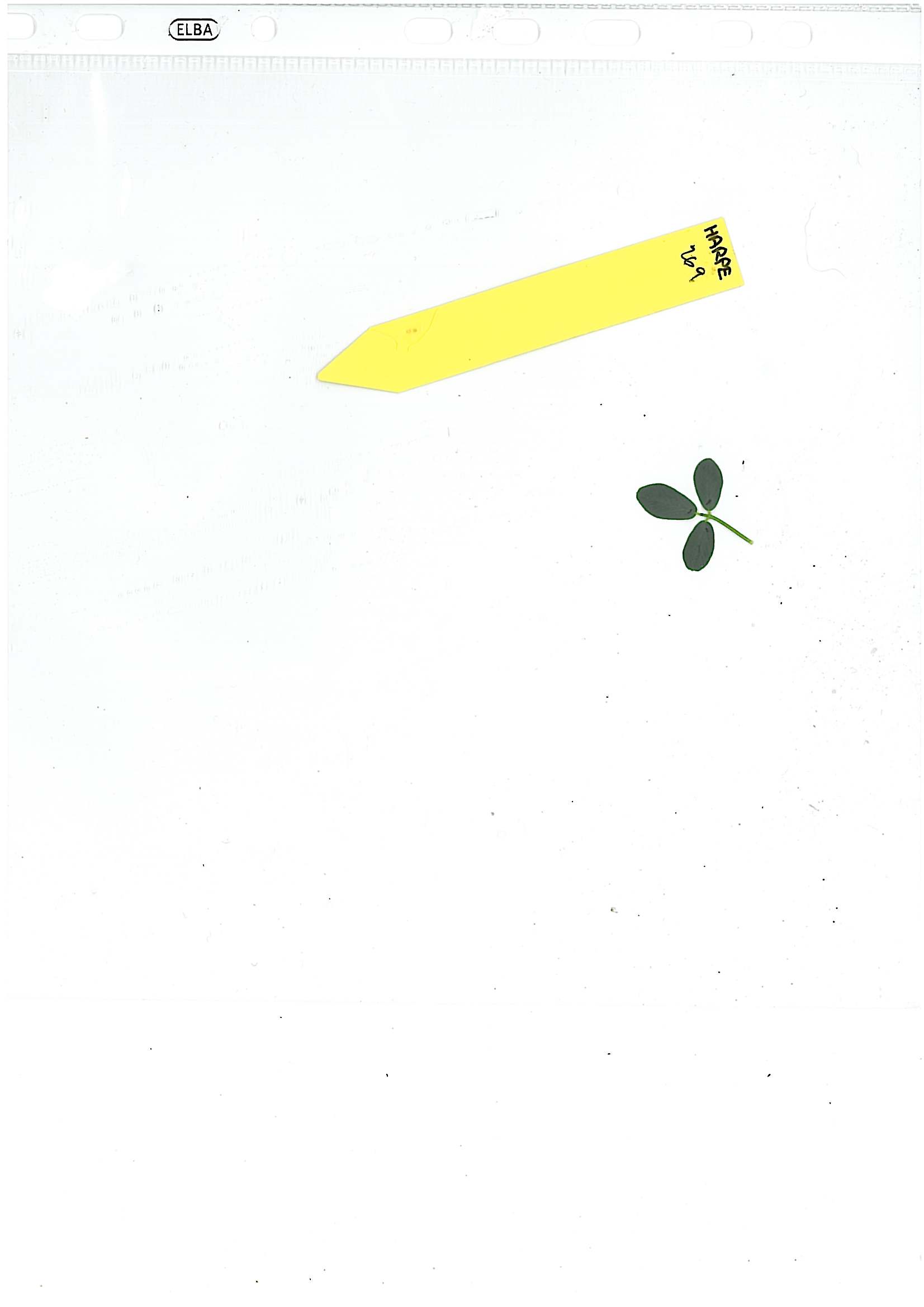** |

Sup. Fig. 1– Alfalfa leaves of Mediterranean (Barmed cv) and temperate (Harpe cv) cultivars grown at different temperatures. Measurements were performed on the youngest mature leaf of the main stem at the end of the experiment (i.e. node ranks 8-9). The horizontal bar represents 1 cm.

Sup. Fig. 2– Comparison of measured and simulated net assimilation rates (µmolCO_2_.m^-2^.s^-1^) for alfalfa leaves from the two temperate (G3, a) and Mediterranean (7_7, b) cuttings studied. Different symbols reflect different growth temperatures.


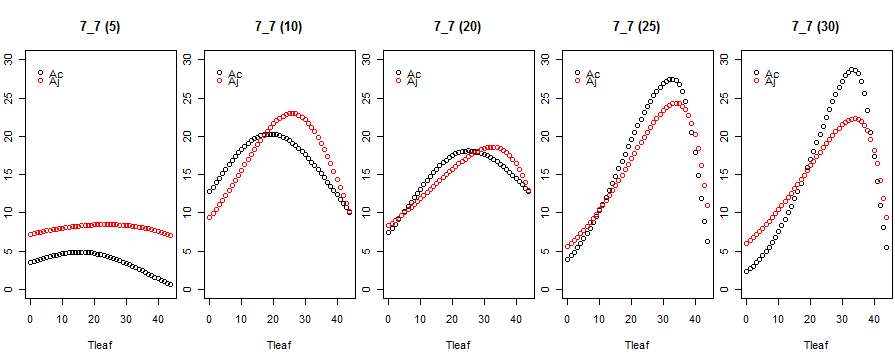


a)

b)

c)

d)

e)


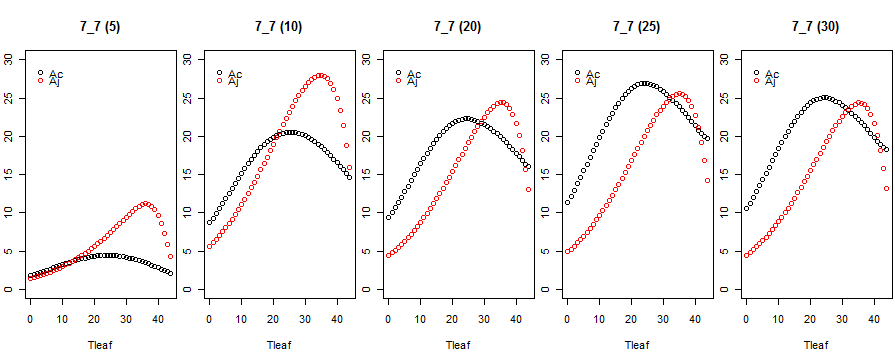


k)

l)

m)

n)

o)


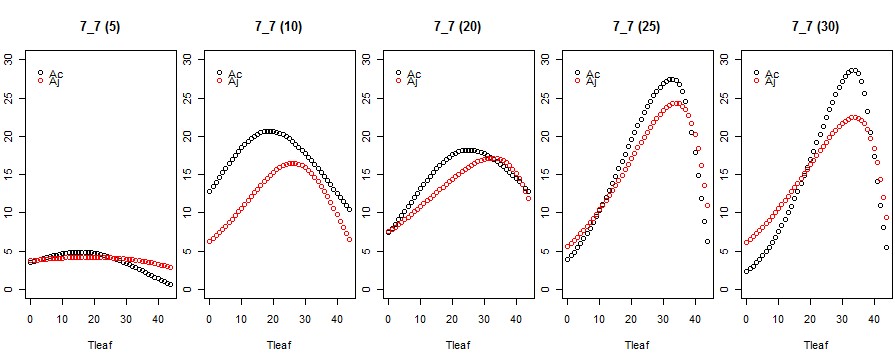


f)

g)

h)

i)

j)

RMSE 0.93

RMSE 1.60

RMSE 2.36

RMSE 3.32

RMSE 2.02

RMSE 1.02

RMSE 2.36

RMSE 2.93

RMSE 3.32

RMSE 2.14

RMSE 1.55

RMSE 3.21

RMSE 3.05

RMSE 3.92

RMSE 3.83

Net Assimilation Rate (µmol.m^-2^.s^-1^)

Leaf Temperature (°C)

Sup. Fig. 3 – Predicted responses to leaf temperature of the RuBP carboxylation limited (black, Ac) and the RuBP regeneration limited (red, Ar) assimilation rates at growth temperatures ranging from 5°C to 35°C under three scenarios: i) using photosynthetic parameters actually measured on the 7_7 cutting and reflecting changes in the J_max_^25^/ V_cmax_ ^25^ ratios and J_max_ and V_cmax_ temperature dependencies (a-e), ii) assuming a constant J_max_^25^/ V_cmax_ ^25^ ratio across growth temperatures (f-j) or iii) assuming unchanged temperature dependencies across growth temperatures (k-o). Arrows indicate the predicted optimal temperature of A_400_.

Sup. Fig. 4 – Impact of growth temperature (T_growth_) on the Photosynthetic Nitrogen Use Efficiency (PNUE) of alfalfa leaves on two cuttings of Mediterranean (filled circles, 7_7 cutting) and temperate (open circles, G3 cutting) origins. PNUE was calculated as the slope of the relationship between the net assimilation rate under standard conditions (i.e. at a leaf temperature of 25°C and 400 ppm CO_2_) and the leaf nitrogen content. The minimum structural N content, represented by the intercept of the relationship with the x axis, was assumed to be constant and fixed at 0.2 g N.m^-2^ according to Louarn et al. (2015).

Sup. Fig. 5 – Impact of growth temperature the maximal light-saturated photosynthesis at T_opt_ (A_400_^opt^) for the two genotypes studied.

Sup. Fig. 6 – Relationship between observed and predicted optimal temperatures. The plain line represents the 1:1 line. The dashed line represent the linear relationship between the two variables (y=0.80x+8.9, r^2^=0.86).

Sup. Table 1 - Parameters for temperature dependencies of the maximum rate of Rubisco carboxylation (*V_cmax_*) and the maximum electron transport rate (*J_max_*) for each growth temperature. Increasing response curves were fitted using Eqn 2, while responses displaying a decline at high leaf temperatures were fitted using Eqn 3. P^25^ stands for the parameter value at 25°C. No fit was possible at

35°C for heat-bleached leaves from the 7_7 genotype.

| Parameter | Genotype | *T_growth_*  (°C) | *P^25^*  (µmol.m^-2^.s^-1^) | *C*  (no unit) | *ΔH_a_*  (J.mol^-1^) | *ΔS*  (J.mol^-1^.K^-1^) | *ΔH_d_*  (J.mol^-1^) |
| --- | --- | --- | --- | --- | --- | --- | --- |
| *V_cmax_* | 7_7 | 5 | 17.1 | 14.304 | 35.323 |  |  |
|  | G3 | 5 | 13.0 | 14.304 | 35.330 |  |  |
|  | 7_7 | 10 | 71.8 | 16.482 | 40.750 |  |  |
|  | G3 | 10 | 95.0 | 19.857 | 49.124 |  |  |
|  | 7_7 | 20 | 77.0 | 21.119 | 52.607 |  |  |
|  | G3 | 20 | 93.6 | 19.173 | 47.613 |  |  |
|  | 7_7 | 25 | 92.8 | 30.887 | 76.594 | 1.008 | 316.542 |
|  | G3 | 25 | 89.6 | 21.262 | 52.455 |  |  |
|  | 7_7 | 30 | 86.1 | 35.274 | 87.447 | 1.014 | 317.459 |
|  | G3 | 30 | 89.3 | 22.027 | 54.294 |  |  |
|  | 7_7 | 35 | - | - | - |  |  |
|  | G3 | 35 | 64.9 | 28.536 | 70.576 |  |  |
|  |  |  |  |  |  |  |  |
| *J_max_* | 7_7 | 5 | 51.5 | 5.484 | 13.276 | 0.534 | 177.548 |
|  | G3 | 5 | 37.2 | 9.561 | 23.360 | 0.637 | 202.896 |
|  | 7_7 | 10 | 175.0 | 16.846 | 41.237 | 0.381 | 117.128 |
|  | G3 | 10 | 195.0 | 7.701 | 18.961 | 0.618 | 199.624 |
|  | 7_7 | 20 | 138.8 | 11.972 | 29.928 | 0.558 | 177.682 |
|  | G3 | 20 | 161.5 | 14.085 | 34.897 | 0.638 | 200.275 |
|  | 7_7 | 25 | 152.6 | 18.321 | 45.418 | 0.976 | 306.518 |
|  | G3 | 25 | 162.3 | 18.957 | 46.938 | 1.272 | 400.839 |
|  | 7_7 | 30 | 140.0 | 16.634 | 41.242 | 0.979 | 307.947 |
|  | G3 | 30 | 148.0 | 18.951 | 46.776 | 1.272 | 400.951 |
|  | 7_7 | 35 | - | - | - | - | - |
|  | G3 | 35 | 95.4 | 28.690 | 70.889 | 0.700 | 218.814 |
